# Chloroplast genomic characterization and phylogenetic analysis of eleven *Persicaria* medicinal plants from Guangxi, Southern China

**DOI:** 10.3389/fpls.2025.1749088

**Published:** 2026-01-22

**Authors:** Zhuxin Li, Yinghan Dai, Shanshan Zhai, Yingying Cao, Junyu Cheng, Meng Ni, Lijuan Song, Junyu Tao, Dapei Zhang, Ziheng Xu, Yongxing Lai, Haibo Tang

**Affiliations:** 1 Guangxi University of Chinese Medicine, Nanning, China; 2 Guangxi Key Laboratory of Translational Medicine for Treating High-Incidence Infectious Diseases with Integrative Medicine, Nanning, China; 3 Guangxi Health Commission Guangxi Key Laboratory of Molecular Biology of Preventive Medicine of Traditional Chinese Medicine, Nanning, China; 4 Guangxi Key Laboratory of Marine Drugs, Nanning, China; 5 Guangxi Vocational University of Agriculture, Nanning, China; 6 Key Laboratory of Characteristic Experimental Animal Models of Guangxi, Nanning, China

**Keywords:** chloroplast genome, Guangxi, medicinal plants, *Persicaria*, phylogenetics

## Abstract

Polygonaceae is widely distributed worldwide, with the genus *Persicaria* being one of its most medicinally important groups. Nonetheless, genomic research on this genus remains relatively sparse. Moreover, accurate identification of *Persicaria* species is challenging due to significant morphological variation and similarities, which hinders the development and utilization of their medicinal resources. To address these issues, this study undertook the sequencing, assembly, and annotation of the chloroplast genomes of 11 *Persicaria* species from Guangxi and reconstructed a phylogenetic tree. The findings reveal that the chloroplast genome lengths of these 11 species range from 159,028 to 161,023 bp, displaying a typical quadripartite structure comprising a large single-copy (LSC) region, a small single-copy (SSC) region, and two inverted repeat (IR) regions. All chloroplast genomes contain four rRNA genes, 30 tRNA genes, and between 74 and 78 protein-coding genes. The codon usage in the chloroplast genomes shows a preference for A or T at the codon endings. The flanking genes at the IR boundaries are highly consistent among the 11 species, indicating that these boundary regions are conserved within the genus *Persicaria*. Phylogenetic analysis indicates that the 11 species form distinct clades within *Persicaria*, separate from the genera Calligonum and Fallopia. The findings of this study reveal significant evolutionary conservation and divergence within *Persicaria* species in Guangxi. The BEAST estimate the stem age of the genus *Persicaria* to be 81.89 Ma. This research elucidates the phylogenetic positions of these 11 species within *Persicaria*, offering a scientific foundation for species identification, advancing the understanding of the evolutionary history of the genus in Guangxi, and providing insights into their genetic diversity to support the rational development and utilization of their medicinal resources.

## 1 Introduction

The genus *Persicaria*, a member of Polygonaceae within the order Caryophyllales, is widely distributed globally, with a predominant presence in the northern temperate regions. *Persicaria* is one of the most important genera with rich medicinal species. In China, more than 13 genera and approximately 269 species are recorded, accounting for over a quarter of the global species and genera (Li, 1998). Polygonaceae are extensively distributed throughout China, with Guangxi Province in the southern part of the country hosting 10 of the 12 genera found in China, representing 83.3% of the national distribution (Wang et al., 2007). These plants exhibit considerable morphological diversity, encompassing herbaceous plants, climbing species, woody vines, shrubs, and trees (Sanchez et al., 2009; Burke and Sanchez, 2011; Schuster et al., 2015). The classification of Polygonaceae, particularly the definition of subfamilies and genera, has long been a subject of debate. Early taxonomic studies largely relied on morphological characteristics for classification. In 1856, Meisner conducted the first comprehensive morphological study of the family, dividing Polygonaceae into four tribes: *Eriogoneae*, *Polygoneae*, *Brunnichieae*, and *Symnerieae*, with *Polygoneae* further subdivided into the *Pterygocarpae* and *Aperocarpae* tribes (Meisner, 1856). In 1978, Haraldson proposed a revised classification based on stem and petiole anatomical features, dividing the family into two subfamilies: Polygonoideae and Eriogonoideae, with the former further subdivided into five tribes: *Polygoneae, Persicarieae, Coccolobeae, Rumiceae*, and *Tripareae* (Haraldson, 1978). The *Flora of China* recognizes two subfamilies within the domestic Polygonaceae: Polygonoideae and Rumicoideae. With the advent of molecular biology techniques, a reclassification of Polygonaceae has been facilitated. As of 2019, the widely accepted classification system includes three subfamilies: Polygonoideae, Eriogoneae, and Symmerioideae (Wang, 2019). Guangxi, with its unique geographical characteristics, is rich in plant diversity, and *Persicaria* are particularly abundant in this region. These plants play a crucial ecological role and possess significant economic and medicinal value. These plants can serve as important components of Chinese herbal feed additives and are widely used in the animal husbandry industry. Not only are they cost-effective, but they also contribute to the green development of animal husbandry and generate positive social benefits. However, due to considerable morphological variation and similarities among species within the family, accurate identification remains a challenge. Therefore, the precise identification of *Persicaria* species is of substantial research significance, providing a theoretical foundation for advancing the understanding of the taxonomy of these plants in Guangxi.

Moreover, the genus *Persicaria* exhibit significant medicinal properties, particularly in Guangxi, where they play a vital role not only in traditional Chinese medicine but also as key constituents of Zhuang and Yao ethnomedicines. The *Illustrated Guide to Zhuang Medicine* documents over 20 medicinal plants within this family, many of which demonstrate therapeutic efficacy. Common pharmacological effects of these plants include heat-clearing, detoxifying, wind-dispelling, dampness-eliminating, and anti-inflammatory activities (Zhu and Dai, 2020). Contemporary research has shown that the chemical constituents of *Persicaria* plants primarily consist of flavonoids, quinones, phenylpropanoids, and terpenoids, which are associated with a wide range of bioactivities, including anti-tumor (Wang et al., 2023), anti-oxidant (Yu et al., 2024), anti-inflammatory, analgesic, antimicrobial, insecticidal, and urinary tract infection treatment effects (Zhu and Dai, 2020; Yang et al., 2023). Previous studies have mainly concentrated on the chemical composition and pharmacological properties of these plants. However, with the development of molecular biology techniques, increasing attention is being given to the genetic basis of *Persicaria* plants, though relevant studies remain limited. Therefore, this study focuses on the investigation of chloroplast genomes in *Persicaria* sp*ecies* species from Guangxi, aiming to provide new insights and a theoretical foundation for further research into the medicinal potential of these plants.

Chloroplasts are vital organelles in plants that facilitate energy conversion and photosynthesis, and are present in terrestrial plants, algae, and certain protists (Asaf et al., 2017a). Beyond their role in photosynthesis, chloroplasts are also involved in the biosynthesis of complex organic compounds, including amino acids and fatty acids (Yao et al., 2015; Guo et al., 2022). The chloroplast genome is typically characterized by a circular, double-stranded DNA structure with a relatively independent genetic sequence (Xing and Liu, 2008; Kan et al., 2024). This genome is distinguished by its small molecular size, slow rate of molecular evolution, high sequence conservation, and low mutation rate (Xing and Liu, 2008; Hu et al., 2022). These properties make chloroplast genomes particularly useful in plant identification, phylogenetic analysis, genetic diversity assessments, and evolutionary studies (Yao et al., 2015; Kan et al., 2024). As such, chloroplast genome sequencing continues to be one of the most reliable methods for reconstructing phylogenetic relationships and elucidating maternal ancestry (Sun et al., 2022; Dong et al., 2025). With the rapid advancements in high-throughput sequencing technologies, the chloroplast genomes of numerous plants, including several key medicinal species, have been sequenced and analyzed. This study focuses on 11 native *Persicaria* sp*ecies.* from Guangxi. Using high-throughput sequencing methods, we sequenced the DNA of these species and subsequently assembled and annotated their chloroplast genomes. Further analysis of the genomic sequence characteristics of the 11 *Persicaria* sp*ecies* from Guangxi were conducted. Using *Fallopia multiflora*, *Calligonum junceum* and *Calligonum klementzii* as the outgroups, a phylogenetic analysis of the chloroplast genome sequences from 53 closely related plant species was performed. This study aimed to determine the phylogenetic position of these species within the genus *Persicaria*, providing a theoretical basis for the identification, development, and utilization of medicinal *Persicaria* species in Guangxi.

## 2 Materials and methods

### 2.1 Plant materials, DNA extraction, and sequencing

The *Persicaria* species, including *Persicaria chinensis*, *Persicaria hydropiper*, *Persicaria capitata*, *Persicaria glabra*, *Persicaria perfoliata*, *Persicaria tinctoria*, *Persicaria longiseta*, and *Persicaria maackiana*, *Persicaria hastatosagittata*, *Persicaria lapathifolia*, and *Persicaria pubescens* were collected from various regions in Guangxi Province, China. Specifically, *P. capitata* (OR730796) and *P. pubescens* (OR730798) were sampled from Wuming District in Nanning, Guangxi; *P. glabra* (OR730797), *P. tinctoria* (OR730799), *P. longiseta* (OR730801), *P. maackiana* (OR730802), *P. hastatosagittata* (OR730803), *P. perfoliata* (OR730805) and *P. lapathifolia* (OR730804) were collected from Shanglin County in Nanning; *P. chinensis* (OR730800) was collected from Xixiangtang District in Nanning; *P. hydropiper* (OR570614) and (OR570615) were respectively collected from Xing’an County in Guilin, Guangxi and Sanjiang County in Liuzhou, Guangxi. The morphological identification of the collected specimens was performed by the Zhuang and Yao Medicinal Herb Laboratory (Guangxi University of Chinese Medicine) and verified by comparison with the Plant Photo Bank of China (PPBC). Specimens of these *Persicaria* species are deposited in the Herbarium of Traditional Chinese Medicine Plants, Guangxi University of Chinese Medicine (GXCM) (**Supplementary Table S1**). For DNA extraction, healthy plant leaves were selected, frozen in liquid nitrogen, and stored at ultra-low temperatures. Genomic DNA was subsequently extracted from the fresh leaf tissue using the hexadecyl trimethyl ammonium bromide (CTAB) method. DNA concentration and purity were assessed using a NanoDrop 2000 spectrophotometer (Thermo Fisher Scientific, USA). DNA samples that met the standards for library construction and fragment length were numbered and sequenced using the Illumina NovaSeq 6000 platform. Sequencing was conducted on an Illumina HiSeq platform by Genesky Biotechnologies Inc. (Shanghai, China). Following sequencing, the raw data were subjected to quality control and filtering using FastQC 0.11.8 version and R 3.6.1 version. The filtering criteria were as follows: (1) sequences containing more than three N bases were discarded; (2) sequences with less than 60% high-quality bases (Phred score ≥20) were removed; (3) low-quality bases at the 3’ end were excluded; (4) sequences shorter than 60 base pairs were discarded. The resulting high-quality sequences (clean reads) were saved in FASTQ format for subsequent assembly and annotation. The chloroplasts genome of *Persicaria* species plants were sequenced with an average depth of 21289 × and a coverage of 100.18%.

### 2.2 Chloroplast genome assembly and annotation

The high-quality sequencing reads obtained were assembled using the metaSPAdes algorithm (Bankevich et al., 2012) to construct the chloroplast genomes of 11 *Persicaria* sp*ecies*, with reference to the previously reported chloroplast genome sequences of *Persicaria* (NCBI accession numbers: NC_050358, NC_058319, NC_067040; **Supplementary Table S1**). The chloroplast genomes of the 11 *Persicaria* species were annotated using CpGAVAS. After chloroplast genomes were predicted and annotated, we manually edited by comparison with the published chloroplast genome sequences (NCBI accession numbers: NC_050358, NC_058319, and NC_067040). The annotated genome sequences have been uploaded to GenBank (https://www.ncbi.nlm.nih.gov/genbank/) with the following accession numbers: OR730796, OR730797, OR730798, OR730799, OR730800, OR730801, OR730802, OR730803, OR730804, OR730805, OR570614, OR570615 (**Table 1**). Finally, the chloroplast genome maps were generated using the OGDRAW 1.1.1 version. The Relative Synonymous Codon Usage (RSCU) analysis was performed using CodonW version 1.4.4 (http://codonw.sourceforge.net/).

**Table 1:** Chloroplast genome features of the 11 Persicaria species.

Species	ALL Genome		LSC		SSC		IR		Location	Accession numbers
	Length	GC%	Length	GC%	Length	GC%	Length	GC%		
Persicaria capitata	160070	37.9	84088	36.1	14164	33.1	30909	41.4	Wuming, Nanning	OR730796
Persicaria glabra	160544	38.1	83572	36.6	14740	33.0	31116	41.5	Shanglin, Nanning	OR730797
Persicaria pubescens	160143	38.2	84351	36.5	13450	33.2	31171	41.5	Wuming, Nanning	OR730798
Persicaria tinctoria	159633	38.2	83725	36.6	13656	33.0	31126	41.5	Shanglin, Nanning	OR730799
Persicaria chinensis	159028	38.0	84348	36.1	12894	33.4	30893	41.4	Xixiangtang, Nanning	OR730800
Persicaria longiseta	159626	38.2	83903	36.5	13471	33.2	31126	41.5	Shanglin, Nanning	OR730801
Persicaria maackiana	161023	37.8	85402	36.1	13395	32.9	31114	41.4	Shanglin, Nanning	OR730802
Persicaria hastatosagittata	160749	37.8	85544	35.9	12955	32.7	31125	41.3	Shanglin, Nanning	OR730803
Persicaria lapathifolia	159351	38.2	83621	36.6	13448	33.2	31141	41.5	Shanglin, Nanning	OR730804
Persicaria perfoliata	160724	38.0	85427	36.1	12927	33.1	31185	41.4	Shanglin, Nanning	OR730805
Persicaria hydropiper^*^	159843	38.2	84350	36.5	13151	33.2	31171	41.5	Xingan, Guilin	OR570614
Persicaria hydropiper^#^	159819	38.2	84351	36.5	13138	33.3	31165	41.5	Sanjing, Liuzhou	OR570615

*The sample was collected from Xing'an County in Guilin (GenBank No. OR570614).

^#^The sample was collected from Sanjiang County in Liuzhou (GenBank No. OR570615).

### 2.3 Repetitive sequences and comparative analysis of the chloroplast genome

Tandem Repeats Finder (Benson, 1999) was used to identify repetitive sequences. Simple Sequence Repeats (SSRs) in the assembled chloroplast genomes were identified using the MISA software. Global alignment analysis of the chloroplast genomes of the 11 *Persicaria* species was performed using the mVISTA software (https://genome.lbl.gov/vista/index.shtml). The inversion (IR) boundaries of these chloroplast genomes were mapped using the IRscope online tool (), and the contraction and expansion of the IR regions were analyzed based on the gene position differences at the boundaries. In order to identify the hypervariable regions within the chloroplast genome of *Persicaria* species, the chloroplast genomes of accessions OR730796 through OR730805 and OR570614 through OR570615 were aligned utilizing MEGA7 software. Subsequent manual adjustments to the alignment were performed using Se-Al version 2.04. The nucleotide diversity (Pi) across the chloroplast genome was then calculated employing DnaSP version 5 software (Librado and Rozas, 2009) through sliding window analysis.

### 2.4 Phylogenetic analysis

To determine the evolutionary relationships within the *Persicaria* genus and clarify the phylogenetic relationships and positions of 11 medicinal species of *Persicaria*, we assembled the chloroplast genomes of the 11 species and included 53 additional chloroplast genome sequences from other Polygonaceae species available in GenBank. These sequences were used to construct a phylogenetic tree for phylogenetic analysis. Orthofinder 2.2.7, with multiple sequence alignment performed using MAFFT, was utilized for the analysis. Phylogenetic trees were constructed using the Maximum Likelihood (ML) method with 1000 bootstrap replicates (Zou et al., 2015), with RAxML-HPC2 7.6.3 (Hu et al., 2022) employed for tree construction.

### 2.5 Estimation of divergence time

To estimate the divergence times among lineages of *Persicaria*, we employed BEAST v1.10.4 (Drummond and Rambaut, 2007) utilizing a concatenated data matrix of complete chloroplast genomes to maximize genus inclusion. The analysis was conducted using the GTR + G substitution model, a relaxed molecular clock model, and the Yule process as the tree prior. In accordance with previous research (Manchester and O’leary, 2010; Yao et al., 2019), two fossil calibrations and two secondary calibrations were incorporated. The crown age of Polygonaceae was constrained to a range of 72.1-66.0 Ma, employing a lognormal calibration prior (Manchester and O’leary, 2010). The crown age of Muehlenbeckia was set within a range of 22.0-19.0 Ma, also using a lognormal calibration prior (Pole, 1992; Schuster et al., 2013). The crown age of Ruprechtia_albida was set within a range of 78.0-76.0 Ma, also using a lognormal calibration prior (Zhang et al., 2022). Both secondary calibration priors were modeled using a normal distribution. The Markov Chain Monte Carlo (MCMC) analysis was executed for 1 x 10^9^ generations, with sampling occurring every 10,000 generations. Convergence of the two independent runs and the stationarity of the chains were assessed using Tracer v1.7 (Rambaut et al., 2018), ensuring that all pertinent parameters exhibited an effective sample size (ESS) greater than 200. The initial 25% of the trees were discarded as burn-in. Subsequently, a maximum clade credibility tree was constructed, reporting mean node heights and 95% highest posterior density intervals (95% HPDs), utilizing TreeAnnotator v1.10.4 (Suchard et al., 2018). The resultant phylogenetic tree was visualized using FigTree v1.3.1.

## 3 Results

### 3.1 Genomic characteristics of chloroplasts

**Figure 1:**
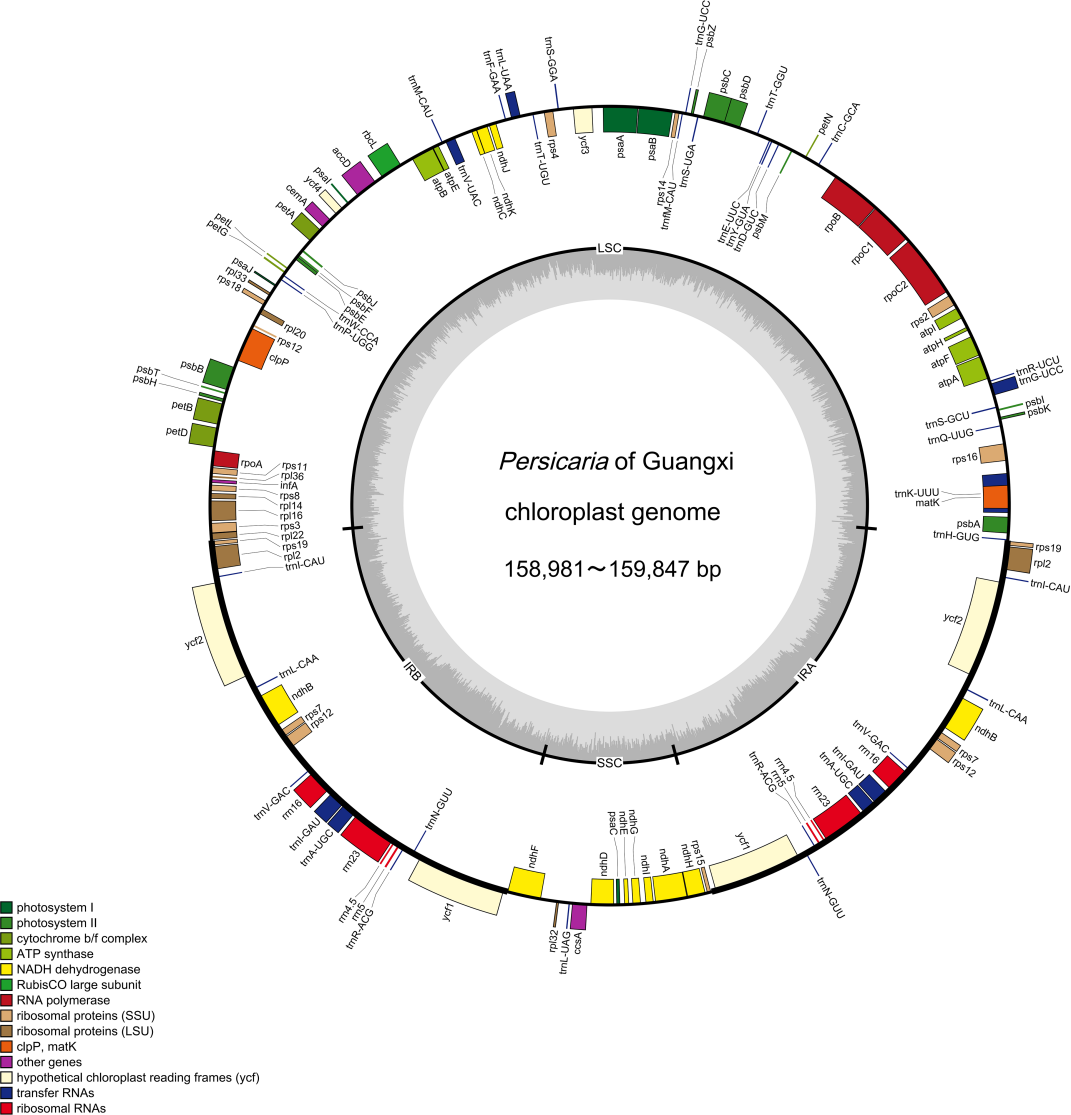
Gene map of the *Persicaria* chloroplast genome. Genes within the circular region are transcribed in a clockwise direction, while those outside the circular region are transcribed in a counterclockwise direction. The inner ring’s dark gray shading represents the GC content, whereas the light gray shading indicates the AT content. Genes are color-coded according to their respective functional categories.

After assembling the clean reads, the length of the chloroplast genomes of 11 *Persicaria* species ranged from 159,028-161,023 bp (**Table 1**; **Figure 1**). Like most angiosperms, the chloroplast genomes of these 11 *Persicaria* species exhibit a typical quadripartite structure, consisting of a large single-copy region (LSC), a small single-copy region (SSC), and two inverted repeat regions (IR) (**Figure 1**). The length of the LSC region for these 11 *Persicaria* species ranged from 83,572-85,544 bp, the SSC region from 12,894-14,740 bp, and the IR regions from 30,893-31,185 bp. The GC content in the LSC, SSC, and IR regions, as well as in the entire chloroplast genome, ranged from 35.9%-36.6%, 32.7%-33.4%, and 41.3%-41.5%, respectively, which is similar to previously reported GC content for chloroplast genomes in *Persicaria* species (**Table 1**).

Through gene annotation, we found that the chloroplast genomes of 11 *Persicaria* species from Guangxi, contained 4 rRNA genes and 30 tRNA genes. The primary differences in the chloroplast genomes were observed in the protein-coding genes. Specifically, *P. capitata* and *P. chinensis* had 74 protein-coding genes, *P. tinctoria* had 75, *P. glabra*, *P. pubescens*, *P. longiseta*, and *P. hydropiper* had 76, *P. perfoliata* had 77, and *P. maackiana*, *P. hastatosagittata*, and *P. lapathifolia* had 78 (**Supplementary Table S2**). With the exception of *P. capitata* and *P. tinctoria*, 18 genes were duplicated across the species, including 7 tRNA genes (*trnI-CAU, trnL-CAA, trnV-GAC, trnI-GAU, trnA-UGC, trnR-ACG, trnN-GUU*), 4 rRNA genes (*rrn16, rrn23, rrn4.5, rrn5*), and 7 protein-coding genes (*ycf2, ycf1, ndhB, rpl2, rps12, rps19, rps7*). Compared to other species, *P. capitata* and *P. chinensis* lacks the *psbL*, *ndhD*, *rpoA*, and rps15 genes, whereas *P. tinctoria* lacks the *psbL*, *ndhD*, and *ycf1* genes. These missing genes were primarily located in the LSC and SSC regions (**Supplementary Table S2**, **Figure 1**). Furthermore, 18 genes contained introns, including 12 protein-coding genes (*rpl16, rpl2, rps12, rps16, rpoC1, ndhB, ndhA, petB, petD, atpF, clpP, ycf3*) and 6 tRNA genes *(trnK-UUU, trnG-UCC, trnL-UAA, trnV-UAC, trnI-GAU, trnA-UGC*). Genes containing introns were mainly distributed in the LSC and IR regions.

### 3.2 Bias of codon usage

Codon usage bias analysis of the chloroplast genomes of 11 *Persicaria* species from Guangxi indicated that the overall size and base composition of the protein-coding regions were nearly identical across the genomes. There were minimal differences in codon usage preferences among the species. The most frequently used codons encoded the amino acid leucine (Leu), appearing 2796, 2853, 2850, 2476, 2799, 2805, 2947, 2929, 2940, 2859, 2920, and 2916 times, respectively. In contrast, the least frequently used codons encoded cysteine (Cys), appearing 297, 302, 298, 267, 297, 295, 305, 305, 305, 302, 299, and 299 times, respectively. Tryptophan (Trp) and methionine (Met) each had a single codon, while other amino acids were represented by 2–6 synonymous codons. Relative Synonymous Codon Usage (RSCU) values greater than 1 indicate a codon preference, values less than 1 indicate lower usage, and RSCU values equal to 1 suggest no preference (Zhong et al., 2023). Most codons ending in A or T exhibited RSCU values greater than 1 (**Supplementary Table S3**), indicating a preference for codons ending in A or T in the chloroplast genomes of these 11 *Persicaria* sp*ecies.*

### 3.3 Long repeats and simple sequence repeats analysis

**Figure 2:**
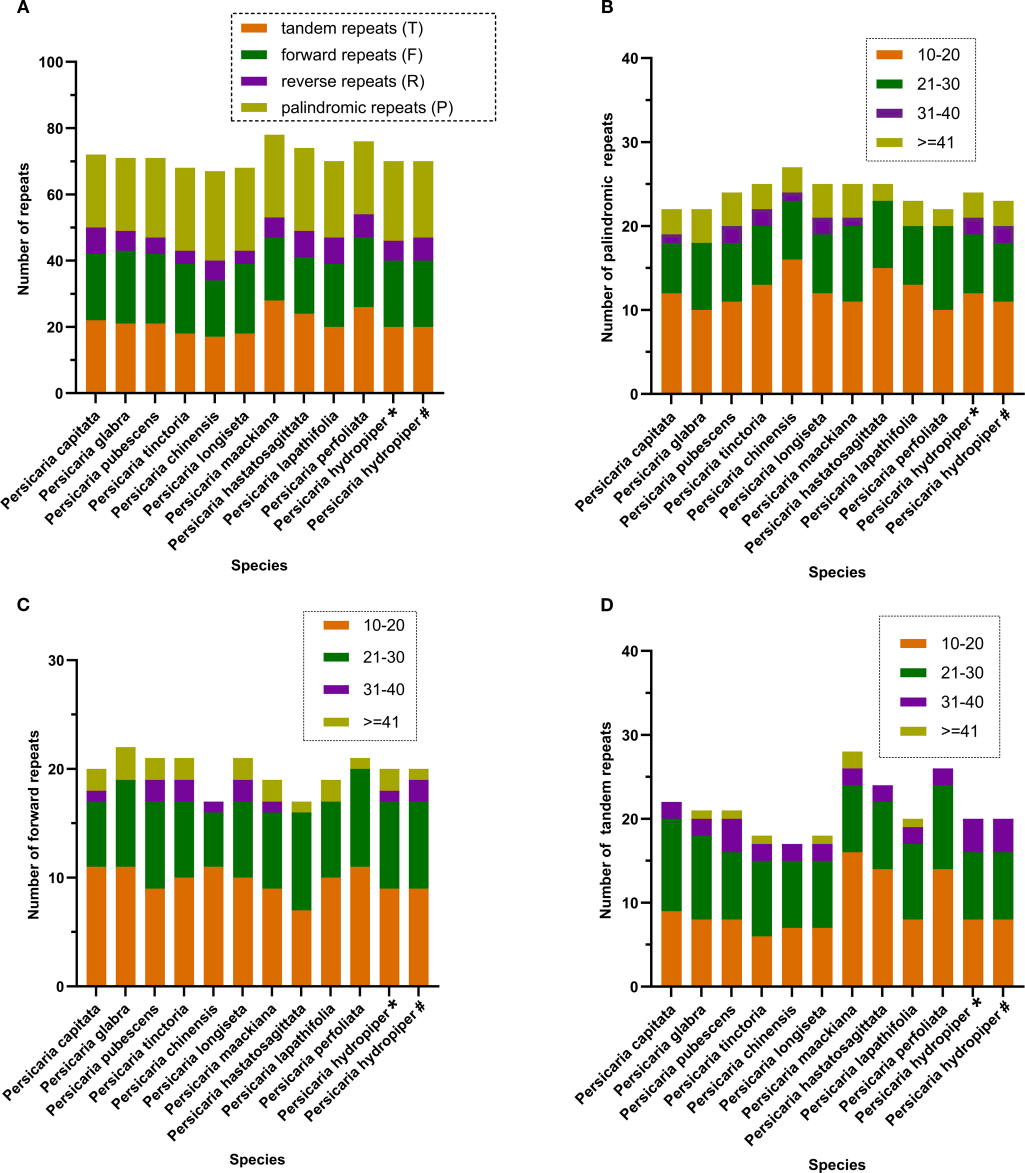
Repeat sequences analysis of the 12 *Persicaria* cp genomes. **(A)** Repeat types in the 12 cp genomes; **(B)** palindromic repeats in the 12 cp genomes; **(C)** forward repeats in the 12 cp genomes; **(D)** tandem repeats in the 12 cp genomes; In **(A)**, different colors show different repeat types; in **(B–D)**, different colors show different lengths. The ordinate represents the number of repeats. *The sample was collected from Xing’an County in Guilin (GenBank No. OR570614). #The sample was collected from Sanjiang County in Liuzhou (GenBank No. OR570615).

Repeated sequences are believed to play an important role in genomic recombination and rearrangement. The repeat sequences in the chloroplast genome include palindromic repeats (P), forward repeats (F), tandem repeats (T), and reverse repeats (R). Our analysis of repeated sequences in the chloroplast genomes of 11 *Persicaria* species from Guangxi revealed that the total number of repeats ranged from 68 to 78. Among these, palindromic repeats ranged from 22 to 27, forward repeats ranged from 17 to 22, tandem repeats ranged from 17 to 28, and reverse repeats ranged from 4 to 8 (**Figure 2**). Furthermore, among the identified repeats, sequences with lengths between 10 and 30 bp were the most abundant. These results are consistent with previously reported findings in other *Persicaria* species, showing a high degree of similarity with the results observed in *P. perfoliata* (Guo et al., 2022; Zeng et al., 2025).

**Figure 3:**
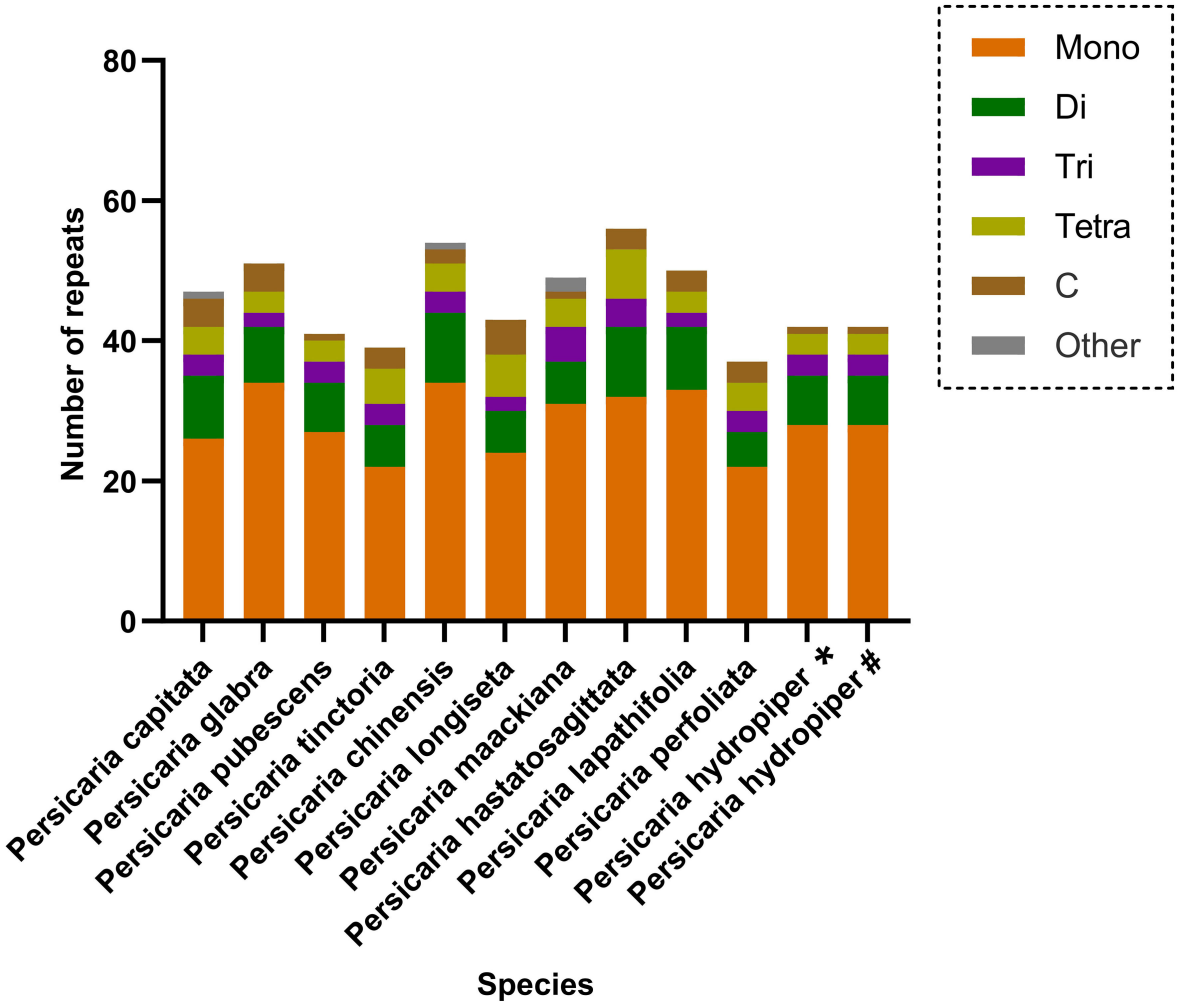
SSR analysis of 12 *Persicaria* cp genomes. Different colors show different repeat types. The ordinate represents the number of repeats. *The sample was collected from Xing’an County in Guilin (GenBank No. OR570614). #The sample was collected from Sanjiang County in Liuzhou (GenBank No. OR570615).

Simple Sequence Repeats (SSRs), or microsatellites, are DNA sequences consisting of short, tandemly repeated nucleotide motifs (1–6 bp in length) that hold significant importance for studies in plant populations (Asaf et al., 2017b). Moreover, chloroplast SSRs exhibiting intraspecific positional variability are commonly utilized as molecular markers for investigating population genetics and evolutionary processes (Yang et al., 2016; Zl et al., 2016). Among the 11 *Persicaria* species investigated in this study, the identified SSRs (excluding those of the ‘c’ type) primarily consisted of mono-, di-, tri-, and tetranucleotide repeat motifs. Mononucleotide repeats were the most abundant, with A/T base compositions being predominant. Dinucleotide repeats were the second most common type, among which AT/TA repeats constituted the majority (**Figure 3**). These findings are consistent with the characteristic pattern observed in angiosperm chloroplast genomes, where SSRs are predominantly composed of polyA and polyT repeats rather than tandem repeats of G/C (Du et al., 2022).

### 3.4 Expansion and contraction of the inverted repeat regions

**Figure 4:**
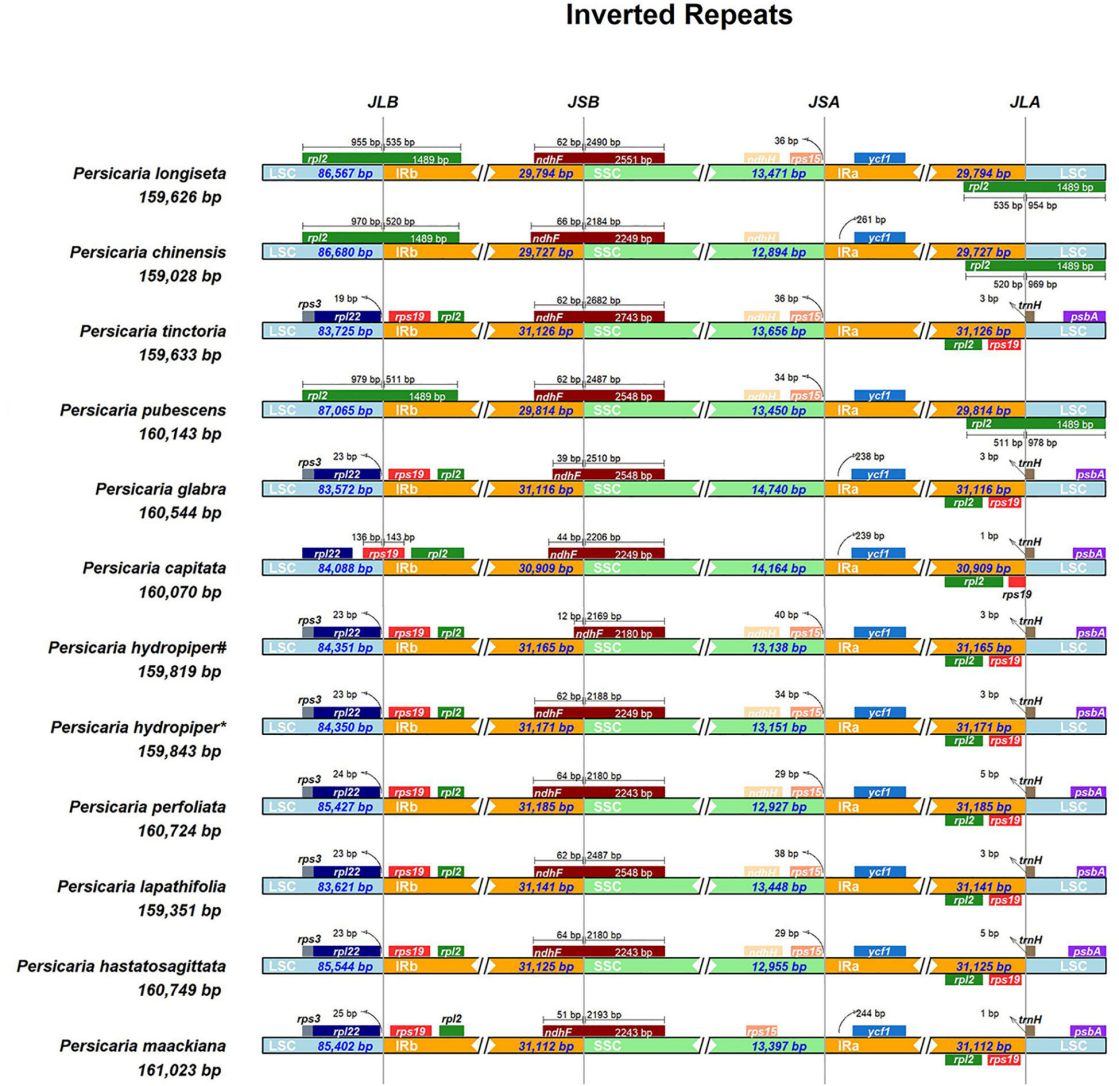
Comparison of the boundaries of LSC, IR, and SSC among chloroplast genomes of 12 cp genomes. JLB, junction between LSC and IRb; JSB, junction between SSC and IRb; JSA, junction between SSC and IRa; JLA, junction between LSC and IRa. *The sample was collected from Xing’an County in Guilin (GenBank No. OR570614). #The sample was collected from Sanjiang County in Liuzhou (GenBank No. OR570615).

Analysis of the expansion and contraction at the inverted repeat (IR) boundaries across the 11 chloroplast genomes of *Persicaria* sp*ecies* from Guangxi revealed highly conserved flanking genes. Specifically, the junctions JLB (LSC-IRb), JSB (SSC-IRb), JSA (SSC-IRa), and JLA (LSC-IRa) were consistent among most species, indicating structural conservation within the family. At the JLB boundary, the flanking genes were *rpl22* and *rps19* in *P. tinctoria*, *P. glabra*, *P. hydropiper*, *P. perfoliata*, *P. lapathifolia*, *P. hastatosagittata*, and *P. maackiana*, with the distance from *rpl22* to the JLB ranging from 19 to 25 bp. In contrast, the JLB boundary was located within the *rpl2* gene in *P. longiseta*, *P. chinensis*, and *P. pubescens*. The expansion into LSC and IRb was 955 bp and 535 bp in *P. longiseta*, 970 bp and 520 bp in *P. chinensis*, and 979 bp and 511 bp in *P. pubescens*, respectively. In *P. capitata*, the JLB was located within *rps19*, with an expansion of 136 bp into LSC and 143 bp into IRb. The JSB boundary was located within the *ndhF* gene in all 11 species. At the JSA boundary, *P. tinctoria*, *P. glabra*, and *P. capitata* had only one flanking gene, either *rps15* or *ycf1*, while the remaining species were flanked by both *rps15* and *ycf1*. At the JLA boundary, the junction was located within *rpl2* in *P. longiseta*, *P. chinensis*, and *P. pubescens*, with expansion into IRa and LSC of 535 bp and 954 bp, 520 bp and 969 bp, and 511 bp and 978 bp, respectively. In the other eight species, the JLA boundary was located within the *trnH* gene, with a variation in distance from *trnH* to the boundary ranging from 1 to 5 bp (**Figure 4**).

### 3.5 Comparative analysis of genomic structure

**Figure 5:**
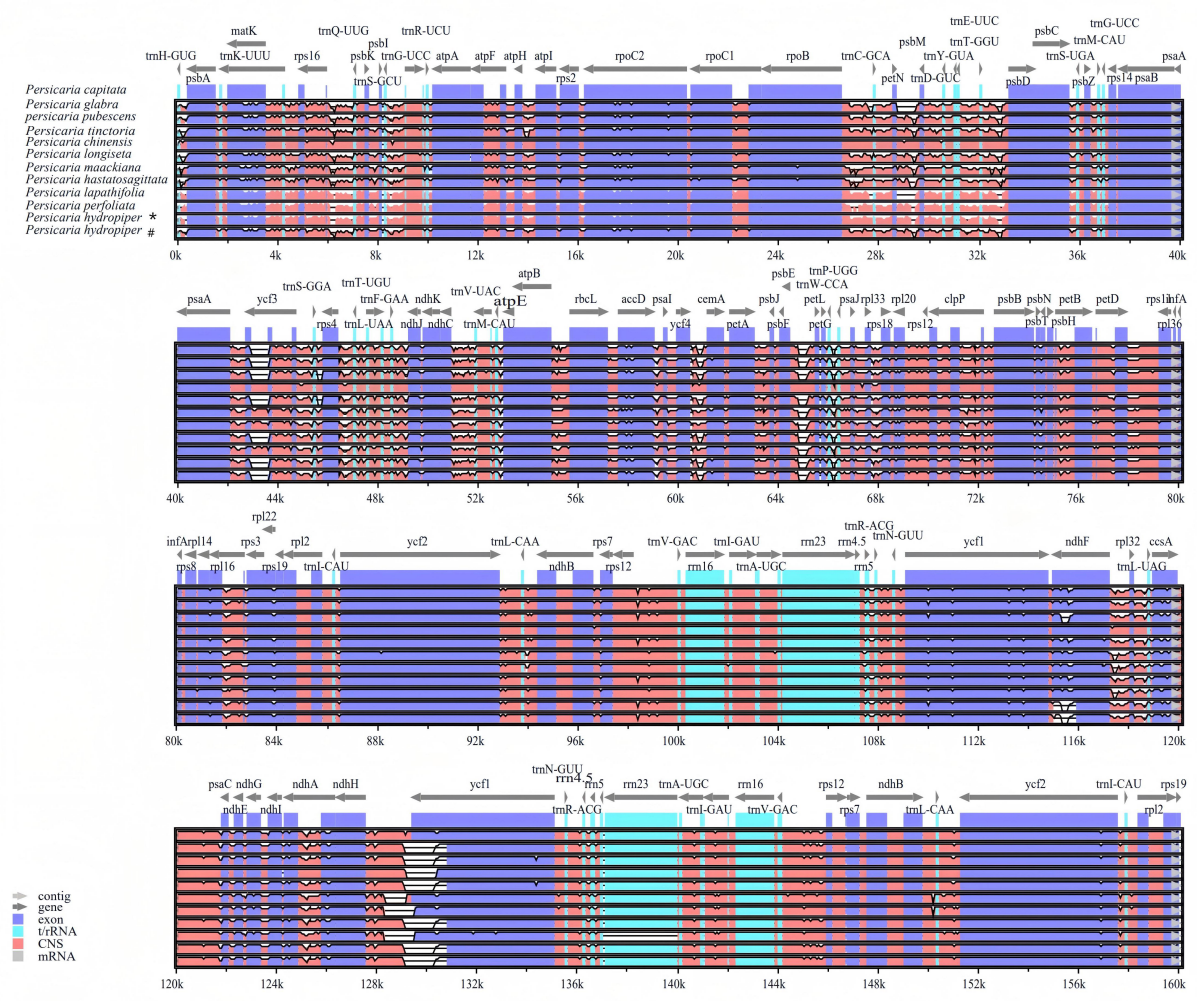
Comparative analysis of chloroplast genome differences in 12 *Persicaria* cp genomes. Gray arrows and thick black lines above the alignment indicate gene orientation. Purple bars represent exons, blue bars represent untranslated regions (UTRs), pink bars represent non-coding sequences (CNS), and gray bars represent mRNA. The y-axis represents the percentage identity (shown: 50–100%). *The sample was collected from Xing’an County in Guilin (GenBank No. OR570614). #The sample was collected from Sanjiang County in Liuzhou (GenBank No. OR570615).

Comparative analysis of chloroplast genomes serves as a crucial component in genomics, enabling the assessment of conservation in gene order and the identification of structural variations among different species. Accordingly, we performed a global alignment of the 11 *Persicaria* chloroplast genomes using mVISTA (**Figure 5**). The results demonstrated that the LSC and SSC regions exhibited greater sequence divergence than the IR (a/b) regions, and non-coding regions were more variable than coding regions. Furthermore, highly divergent regions in these 11 *Persicaria* species were primarily distributed in intergenic spacers.

**Figure 6:**
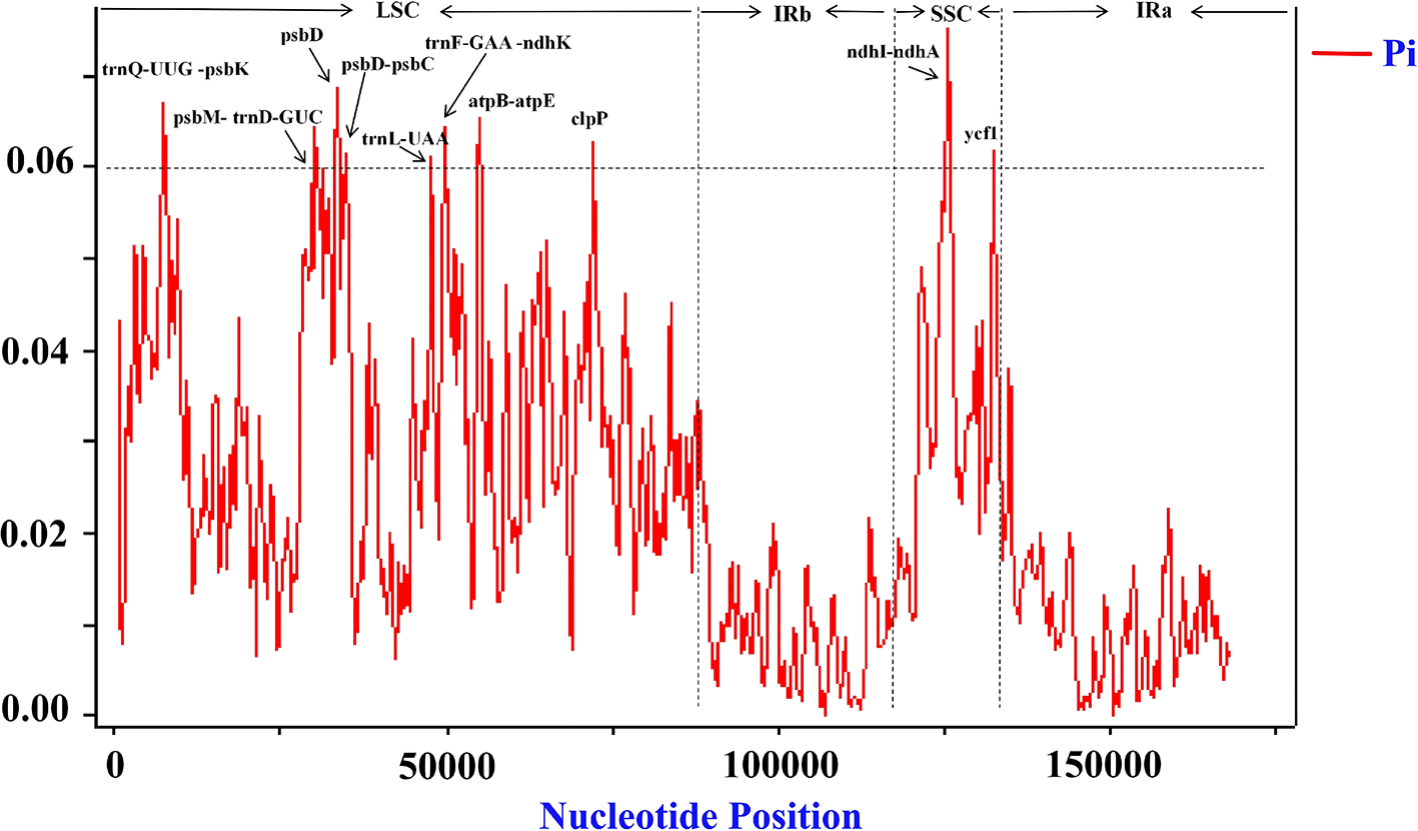
Nucleotide diversity (Pi) among cp genomes of 12 *Persicaria* cp genomes.

To identify highly variable regions, we conducted an analysis of variable sites within the 11 *Persicaria* chloroplast genomes using a sliding window approach implemented in the software DnaSP. The divergent loci, specifically *trnQ-UUG-psbK, psbM-trnD-GUC, psbD, psbD-psbC, trnL-UAA, trnF-GAA-ndhK, atpB-atpE, clpP, ndhI-ndhA* and *ycf1*, exhibited a nucleotide diversity (pi) value of 0.06 or higher (**Figure 6**). The *trnQ-UUG-psbK, psbM-trnD-GUC, psbD, psbD-psbC, trnL-UAA, trnF-GAA-ndhK, atpB-atpE* and *clpP* loci were located in the LSC region, whereas the *ndhI-ndhA* and *ycf1* were situated in the SSC region; no divergent loci were identified in the IR region. These findings corroborate the observation that the LSC and SSC regions are less conserved than the IR regions. The ten genes *trnQ-UUG-psbK, psbM-trnD-GUC, psbD, psbD-psbC, trnL-UAA, trnF-GAA-ndhK, atpB-atpE, clpP, ndhI-ndhA* and *ycf1* were identified as highly divergent and may serve as potential molecular markers for phylogenetic analyzes.

### 3.6 Phylogenetic analysis

**Figure 7:**
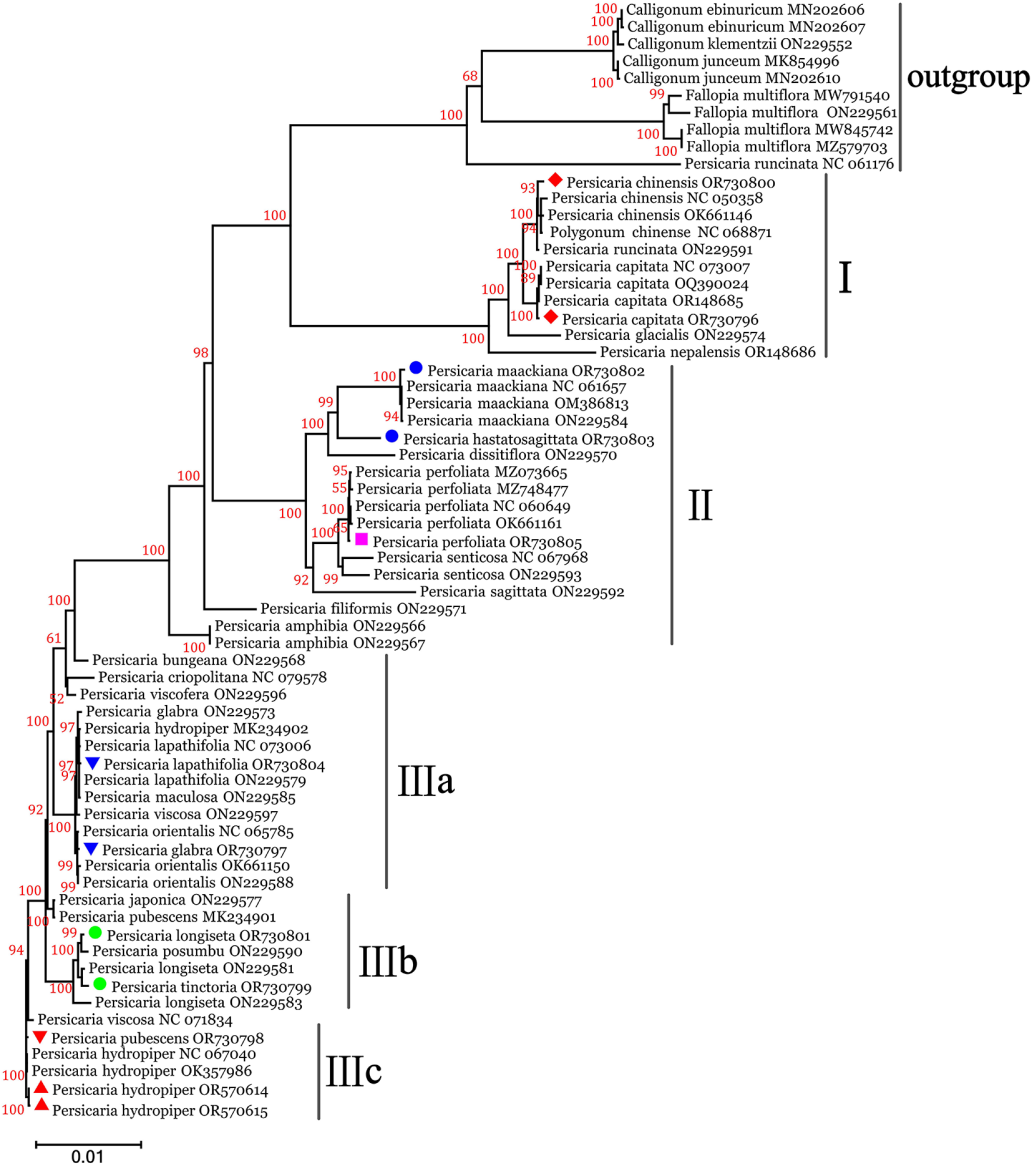
Phylogenetic tree reconstruction containing the chloroplast genomes from the 11 studied species and 53 additional Polygonaceae species based on Maximum Likelihood (ML) analysis. The numerical values on the branches indicate the bootstrap support (BS) values.

To further elucidate the phylogenetic relationships among these 11 *Persicaria* species and clarify their taxonomic positions at the genomic level, a phylogenetic tree was reconstructed using the chloroplast genome sequences of these 11 species along with 53 other Polygonaceae species (**Figure 7**). The results demonstrated that all 11 species from Guangxi cluster within the genus *Persicaria*, forming distinct clades separate from the genera *Calligonum* and *Fallopia*. The *Persicaria* species in Guangxi can be further classified into three groups. *P. chinensis* and *P. capitata* (clade I) are perennials grown for their stingless foliage distinct from other species perhaps representing synapomorphies. *P. perfoliata*, *P. maackiana* and *P. hastatosagittata* are annual plants and their leaves are armed with spines. These characteristics are different from those of other species. Evolutionary relationships show that it belongs to clade II. Regarding their relationships, *P. hydropiper*, *P. pubescens*, *P. tinctoria*, *P. longiseta*, *P. glabra*, and *P. lapathifolia* exhibited closer phylogenetic affinities. This group of plants is classified into clade III groups. *P. lapathifolia* and *P. glabra* have more similarities in morphology, and their genetic evolutionary trees show that they are also more closely related. They belong to the clade IIIa. Similarly, *P. longiseta*, and *P. tinctoria* formed a closely related subgroup IIIb, while *P. pubescens* and *P. hydropiper* showed a sister relationship. This confirms that chloroplast genomic data can serve as a reliable tool for species identification and helps to address the gap in species discrimination within the *Persicaria* species using chloroplast genomes.

### 3.7 Time estimation analysis

**Figure 8:**
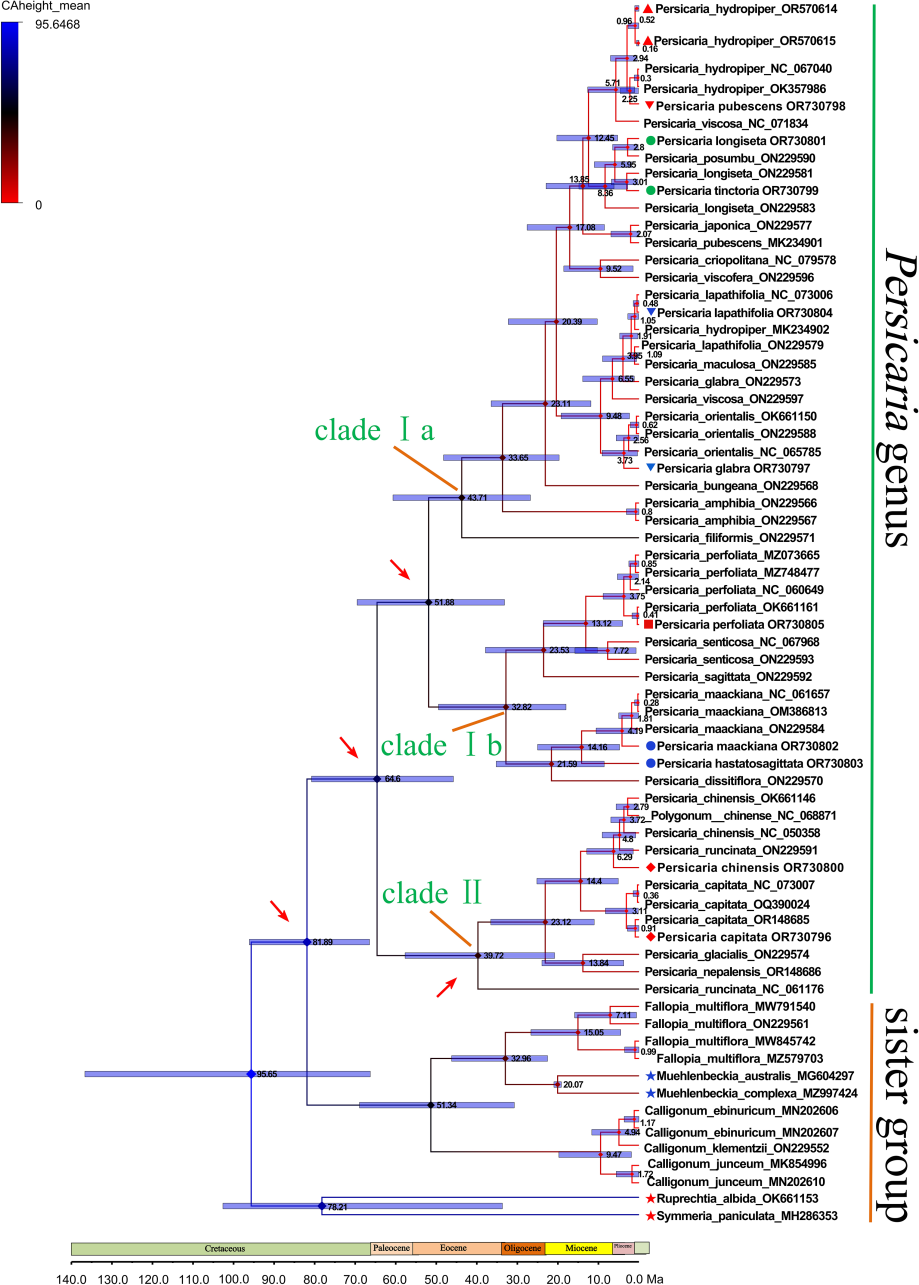
A BEAST-derived chronogram of the Polygonaceae family, based on complete chloroplast genomes, incorporates two fossil calibrations (indicated by blue pentagrams) and two secondary calibration points (indicated by red pentagrams). The fossil calibration for *Muehlenbeckia* is set within a range of 22.0-19.0 Ma, utilizing a lognormal calibration prior (Pole., 1992; Schuster et al., 2013). Similarly, the crown age of *Ruprechtia albida* is calibrated within a range of 78.0-76.0 Ma, also employing a lognormal calibration prior, as reported (Zhang et al., 2022). The numbers above the tree branches represented the mean divergence ages, while the blue bars represented the 95% highest posterior density (HPD) confidence intervals for the node ages (Ma).

The results of the dating analyzes indicate that the *Persicaria* genus diverged from other members of the Polygonaceae family during the late Cretaceous epoch, approximately 81.89 million years ago (Ma), with a 95% highest posterior density (HPD) interval of 66.71 to 96.72 Ma. The two main clades within the *Persicaria* genus, designated as Clade I and Clade II, diverged approximately 64.6 Ma (95% HPD: 43.98-83.74 Ma). Clade I, which includes *Persicaria hydropiper* and *Persicaria filiformis*, likely diverged around 51.88 Ma (95% HPD: 32.67-72.77 Ma). The stem node of *Persicaria filiformis* and *Persicaria amphibia* indicates that it diverged from its sister clades during the Eocene epoch, with a minimum age of 43.71 Ma (26.4-61.08 Ma) (**Figure 8**). In Guangxi, *Persicaria* species are predominantly found within Clade I. The stem node of Clade Ib, which includes *Persicaria hastatosagittata* and *Persicaria maackiana*, diversified approximately 32.82 Ma (18.01-49.45 Ma). Within Clade II, which includes the *Persicaria chinensis* and *Persicaria capitata* diverged during the Eocene epoch, approximately 39.72 Ma (20.79-57.7 Ma). The stem node of the Clade II is a little earlier time frame to the clade Ib diversified around 32.82 (18.01-49.45) Ma (**Figure 8**).

## 4 Discussion

Due to its unique geographical location, Guangxi possesses abundant resources of Polygonaceae plants, particularly the genus *Persicaria*, many of which, such as *P. hydropiper* and *P. chinensis*, hold significant medicinal and economic value. In this study, we sequenced, assembled, annotated, and conducted comparative analysis of the chloroplast genomes of 11 *Persicaria* species from Guangxi: *P. hydropiper*, *P. pubescens*, *P. tinctoria*, *P. longiseta*, *P. glabra*, *P. lapathifolia*, *P. perfoliata*, *P. hastatosagittata*, *P. maackiana*, *P. capitata* and *P. chinensis*. The chloroplast genomes of angiosperms typically range from 120 to 180 kb in size and encode 100–200 genes, including 70–80 protein-coding genes, 30–32 tRNA genes, and 4 rRNA genes (Li et al., 2017). Consistent with most angiosperms, the chloroplast genomes of these 11 *Persicaria* species range in length from 159,028 to 161,023 bp and exhibit the typical quadripartite structure, comprising a large single-copy (LSC) region, a small single-copy (SSC) region, and a pair of inverted repeat (IR) regions. All 11 chloroplast genomes contain 4 rRNA genes and 30 tRNA genes. Variation among these genomes was primarily observed in the number of protein-coding genes: *P. capitata* possesses 74, *P. tinctoria* has 75, *P. glabra*, *P. pubescens*, *P. longiseta* and *P. hydropiper* contain 76 each, *P. perfoliata* has 77, while *P. maackiana*, *P. hastatosagittata* and *P. lapathifolia* possess 78 protein-coding genes. The variation in protein-coding gene numbers is likely attributed to genomic alterations, such as gene mutations or deletions, which may have occurred during the evolutionary adaptation of these species to different geographic environments. Although the examined species exhibit morphological similarities, close phylogenetic relationships, and largely conserved protein-coding gene repertoires—as supported by synteny and phylogenetic tree analyzes—minor differences in gene counts were observed across populations from distinct regions. The molecular basis underlying this geographical divergence in gene content, however, requires further investigation.

Furthermore, analysis of genomic characteristics revealed that the GC content of the LSC, SSC, IR regions, and the complete chloroplast genome ranged from 35.9% to 36.6%, 32.7% to 33.4%, 41.3% to 41.5%, and 37.8% to 38.2%, respectively. These values are consistent with those reported for other *Persicaria* species (**Table 1**). GC content serves as a significant indicator for elucidating phylogenetic relationships among species (Guo et al., 2022). The 11 *Persicaria* species investigated in this study exhibited comparable GC content, with the IR regions demonstrating a higher GC content than the LSC and SSC regions. This phenomenon, which is commonly observed in other plant species (Guo et al., 2018; Liang et al., 2019), indicates a high degree of genetic conservation and close phylogenetic relationships among the 11 *Persicaria* species studied. Understanding the predominant codons used for amino acid encoding, i.e., analyzing codon usage bias (CUB), can significantly enhance the expression efficiency of relevant genes in processes such as genetic breeding (Yuan et al., 2020). Different species exhibit distinct preferences in the usage of synonymous codons, a phenomenon resulting from long-term co-evolution and adaptation between plants and their environments (Suzuki, 2010). Based on this, we investigated the codon usage bias in 11 *Persicaria* species from Guangxi. The overall size and nucleotide composition of the protein-coding regions were largely consistent across these species. The chloroplast genomes showed minimal divergence in codon usage patterns, with a strong preference for A/T endings. This indicates a pronounced bias towards A/T-ending codons, which is consistent with findings in other species within the genus *Persicaria*, suggesting a conserved codon usage pattern among *Persicaria* species. Furthermore, this result implies close phylogenetic relationships and limited genetic divergence among the 11 studied species, which aligns with the observations from the chloroplast GC content analysis.

Long repeats sequences and simple sequence repeats (SSRs) play crucial roles in the identification of germplasm resources, as well as in studies of plant genetic evolution, population genetic polymorphism, and genetic diversity in higher plants (Hu et al., 2022; Akkak et al., 2009; Yu et al., 2013). In the 11 *Persicaria* species from Guangxi, the following repeats were detected: 17–28 tandem repeats, 17–22 forward repeats, 4–8 reverse repeats, and 22–27 palindromic repeats (**Figure 2**). Among the identified SSRs in these species, excluding those of the complex (c) type, the majority consisted of mono-, di-, tri-, and tetranucleotide motifs. Mononucleotide repeats were the most abundant, predominantly composed of A/T bases, followed by dinucleotide repeats, which were primarily comprised of AT/TA motifs. This pattern is consistent with the characteristic composition of angiosperm chloroplast SSRs, which are mainly composed of polyA and polyT repeats rather than G/C tandem repeats (Du et al., 2022). These findings provide a molecular foundation for further research on genetic evolution and molecular marker development in Persicaria species.

Contraction and expansion of the inverted repeat (IR) regions are widespread phenomena in the chloroplast genomes of green plants, serving as a major factor contributing to variations in genome size and structural diversity (Xue et al., 2019). Synteny analysis of chloroplast genomes represents a critical component of genomic studies, enabling the comparison of gene order conservation and the identification of structural variations among different species. In this study, analysis of the 11 *Persicaria* species revealed highly conserved flanking genes at the IR boundaries, with the IR regions ranging between 30 and 32 kb in length. Longer IR regions have been proposed to reduce the impact of structural rearrangements, thereby contributing to higher evolutionary conservation of the chloroplast genome (Palmer and Thompson, 1981). Combined with previous reports on IR boundaries in *Persicaria*, these findings indicate that the boundary regions are highly conserved within the genus *Persicaria*, while conservation across different genera of Polygonaceae requires further verification with more intergeneric data. Contraction analysis further demonstrated that the LSC and SSC regions exhibited greater sequence divergence than the IR (a/b) regions, and non-coding regions were more variable than coding regions. This indicates high evolutionary stability and strong conservation of the chloroplast genome during plant evolution. Additionally, highly divergent regions in these 11 *Persicaria* species were predominantly located in intergenic spacers. This observation is consistent with the patterns observed in the contraction and expansion of the boundary regions.

Phylogenetic trees constructed based on chloroplast genome sequences are instrumental in elucidating genetic evolutionary relationships among species. In the phylogenetic tree generated in this study, the 11 *Persicaria* species from Guangxi demonstrated high consistency with previously published genomic sequences of corresponding species, confirming the utility of chloroplast genomic data for accurate species identification at the molecular level. This finding addresses a critical gap in the application of chloroplast genomes for species discrimination within the family. The classification and taxonomic placement of these 11 species, inferred from chloroplast genomic data, exhibit strong congruence with traditional morphological classifications (Li, 1998), thereby providing a robust molecular foundation for genus- and species-level taxonomy and highlighting the advantage of chloroplast genomes in phylogenetic systematics. The phylogenetic tree, reconstructed using chloroplast genomes from the 11 studied species and 53 additional Polygonaceae species (as outgroups or reference taxa) obtained from the NCBI database, clearly indicates that all Guangxi species cluster within the genus *Persicaria*, forming distinct clades separate from the genera Calligonum and Fallopia. Regarding phylogenetic affinities within *P. hydropiper*, *P. pubescens*, *P. tinctoria*, *P. longiseta*, *P. glabra*, and *P. lapathifolia* form a closely related group. Similarly, *P. perfoliata*, *P. hastatosagittata*, and *P. maackiana* exhibit a sister relationship, and *P. capitata* and *P. chinensis* also show close evolutionary ties. In recent years, advances in high-throughput sequencing and bioinformatics have enabled extensive characterization of chloroplast genomes in medicinal plants. The chloroplast genome serves as a robust super-barcode for species discrimination, especially among phylogenetically close taxa, as demonstrated by the sequencing of 11 *Persicaria* species from Guangxi. Whole-chloroplast genome analyzes provide molecular corroboration of these taxonomic and functional distinctions (Wang, 2019). However, the precise phylogenetic relationships among these 11 species require further validation through integrative analysis incorporating morphological, anatomical, cytological, and additional molecular biological data.

Our BEAST analysis focused on estimating the divergence times within the sampled lineages. The results indicate that the stem age of the genus *Persicaria* is approximately 81.89 Ma (**Figure 8**). While this estimate suggests an early divergence for the lineage leading to *Persicaria*, we acknowledge that our sampling was concentrated on this genus. Therefore, unlike previous studies that inferred family-level divergence times using broad generic sampling (e.g., 55.8–70.6 Ma or 110.9 Ma for Polygonaceae) (Schuster et al., 2013; Zhang et al., 2022). Our results specifically reflect the evolutionary history of *Persicaria* species in Guangxi. The discrepancy with some family-level estimates may be attributed to differences in taxonomic sampling density and calibration strategies.

## 5 Conclusion

Through comprehensive analysis of the chloroplast genomes of 11 medicinal *Persicaria* species from Guangxi, this study reveals high evolutionary conservation and close phylogenetic relationships among these plants. Genomic evidence clarifies the taxonomic placement of the 11 species within the genus *Persicaria*. The precise phylogenetic positioning of these species within the family provides a scientific basis for: accurate species identification of medicinal *Persicaria* in Guangxi; enhanced understanding of the evolutionary history of *Persicaria* in this region; and insights into genetic diversity and differentiation within these species. Furthermore, these findings will facilitate resolving intra-generic phylogenetic relationships of *Persicaria* and support the discovery of new medicinal resources within the genus.

## Data Availability

The datasets presented in this study can be found in online repositories. The names of the repository/repositories and accession number(s) can be found in the article/**Supplementary Material**.
