# Supplementary material for: Chloroplast genomic characterization and phylogenetic analysis of eleven Persicaria medicinal plants from Guangxi, Southern China
Source: Front Plant Sci. 2026 Jan 22;16:1749088. doi: 10.3389/fpls.2025.1749088 (PMC12872838; doi:10.3389/fpls.2025.1749088)
Supplement: Supplementary file 1 [file Table1.docx]

Table S1 Sample information of the 11 *Persicaria* species

| No. | Species | Voucher No. | Location | Reference |
| --- | --- | --- | --- | --- |
| 1 | Persicaria capitata | THL | Wuming, Nanning | NC_050358 |
| 2 | Persicaria glabra | gl | Shanglin, Nanning |  |
| 3 | Persicaria pubescens | FM | Wuming, Nanning |  |
| 4 | Persicaria tinctoria | Shui1 | Shanglin, Nanning |  |
| 5 | Persicaria chinensis | HTM | Xixiangtang, Nanning |  |
| 6 | Persicaria longiseta | Chz1 | Shanglin, Nanning |  |
| 7 | Persicaria maackiana | Chjy1 | Shanglin, Nanning | NC_058319 |
| 8 | Persicaria hastatosagittata | Jy1 | Shanglin, Nanning |  |
| 9 | Persicaria lapathifolia | Shmy1 | Shanglin, Nanning |  |
| 10 | Persicaria perfoliata | kbg | Shanglin, Nanning |  |
| 11 | Persicaria hydropiper^*^ | Bch | Xingan, Guilin | NC_067040 |
| 12 | Persicaria hydropiper^#^ | Sj | Sanjing, Liuzhou |  |

*The sample was collected from Xing'an County in Guilin (GenBank No. OR570614)

#The sample was collected from Sanjiang County in Liuzhou (GenBank No. OR570615)
