# Supplementary material for: Chloroplast genomic characterization and phylogenetic analysis of eleven Persicaria medicinal plants from Guangxi, Southern China
Source: Front Plant Sci. 2026 Jan 22;16:1749088. doi: 10.3389/fpls.2025.1749088 (PMC12872838; doi:10.3389/fpls.2025.1749088)
Supplement: Supplementary file 2 [file Table2.docx]

Table S2 Encoded genes in chloroplast genome of the 11 *Persicaria* species

| Category | Group | Genes | | | | | | | | | | | |
| --- | --- | --- | --- | --- | --- | --- | --- | --- | --- | --- | --- | --- | --- |
|  |  | *P. capitata* | *P. glabra* | *P. pubescens* | *P. tinctoria* | *P. chinensis* | *P. longiseta* | *P. maackiana* | *P. hastatosagittata* | *P. lapathifolia* | *P. perfoliata* | *P. hydropiper** | *P. hydropiper#* |
| self-replication | Large subunit of ribosomal | rpl33, rpl20,  rpl36, rpl14, rpl16^a^, rpl22, rpl2^ac^, rpl32 | rpl33, rpl20, rpl36, rpl14, rpl16^a^, rpl22, rpl2^ac^, rpl32 | rpl33, rpl20, rpl36, rpl14, rpl16^a^, rpl22, rpl2^ac^, rpl32 | rpl33, rpl20, rpl36, rpl14, rpl16^a^, rpl22, rpl2^ac^, rpl32 | rpl33, rpl20, rpl36, rpl14, rpl16^a^, rpl22, rpl2^ac^, rpl32 | rpl33, rpl20, rpl36, rpl14, rpl16^a^, rpl22, rpl2^ac^, rpl32 | rpl33, rpl20, rpl36, rpl14, rpl16^a^, rpl22, rpl2^ac^, rpl32 | rpl33, rpl20, rpl36, rpl14, rpl16^a^, rpl22, rpl2^ac^, rpl32 | rpl33, rpl20, rpl36, rpl14, rpl16^a^, rpl22, rpl2^ac^, rpl32 | rpl33, rpl20, rpl36, rpl14, rpl16^a^, rpl22, rpl2^ac^, rpl32 | rpl33, rpl20, rpl36, rpl14, rpl16^a^, rpl22, rpl2^ac^, rpl32 | rpl33, rpl20, rpl36, rpl14, rpl16^a^, rpl22, rpl2^ac^, rpl32 |
|  | Samll subunit of ribosomal | rps12^ac^, rps16^a^, rps2, rps14, rps4, rps18, rps11, rps8, rps3, rps19^c^, rps7^c^ | rps12^ac^, rps16^a^, rps2, rps14, rps4, rps18, rps11, rps8, rps3, rps19^c^, rps7^c^, rps15 | rps12^ac^, rps16^a^, rps2, rps14, rps4, rps18, rps11, rps8, rps3, rps19^c^, rps7^c^, rps15 | rps12^ac^, rps16^a^, rps2, rps14, rps4, rps18, rps11, rps8, rps3, rps19^c^, rps7^c^, rps15 | rps12^ac^, rps16^a^, rps2, rps14, rps4, rps18, rps11, rps8, rps3, rps19^c^, rps7^c^ | rps12^ac^, rps16^a^, rps2, rps14, rps4, rps18, rps11, rps8, rps3, rps19^c^, rps7^c^, rps15 | rps12^ac^, rps16^a^, rps2, rps14, rps4, rps18, rps11, rps8, rps3, rps19^c^, rps7^c^, rps15 | rps12^ac^, rps16^a^, rps2, rps14, rps4, rps18, rps11, rps8, rps3, rps19^c^, rps7^c^, rps15 | rps12^ac^, rps16^a^, rps2, rps14, rps4, rps18, rps11, rps8, rps3, rps19^c^, rps7^c^, rps15 | rps12^ac^, rps16^a^, rps2, rps14, rps4, rps18, rps11, rps8, rps3, rps19^c^, rps7^c^, rps15 | rps12^ac^, rps16^a^, rps2, rps14, rps4, rps18, rps11, rps8, rps3, rps19^c^, rps7^c^, rps15 | rps12^ac^, rps16^a^, rps2, rps14, rps4, rps18, rps11, rps8, rps3, rps19^c^, rps7^c^, rps15 |
|  | Subunits of RNA polymerase | rpoC2, rpoC1^a^, rpoB | rpoC2, rpoC1^a^, rpoB, rpoA | rpoC2, rpoC1^a^, rpoB, rpoA | rpoC2, rpoC1^a^, rpoB, rpoA | rpoC2, rpoC1^a^, rpoB | rpoC2, rpoC1^a^, rpoB, rpoA | rpoC2, rpoC1^a^, rpoB, rpoA | rpoC2, rpoC1^a^, rpoB, rpoA | rpoC2, rpoC1^a^, rpoB, rpoA | rpoC2, rpoC1^a^, rpoB, rpoA | rpoC2, rpoC1^a^, rpoB, rpoA | rpoC2, rpoC1^a^, rpoB, rpoA |
|  | tRNA | trnH-GUG, trnK-UUU^a^, trnQ-UUG, trnS-GCU, trnG-UCC^a^, trnR-UCU, trnC-GCA, trnD-GUC, trnY-GUA, trnE-UUC, trnT-GGU, trnS-UGA, trnM-CAU  trnS-GGA, trnT-UGU, trnL-UAA^a^, trnF-GAA, trnV-UAC^a^, trnW-CCA, trnP-UGG, trnI-CAU^c^, trnL-CAA^c^, trnV-GAC^c^, trnI-GAU^ac^, trnA-UGC^ac^, trnR-ACG^c^, trnN-GUU^c^, trnL-UAG, trnG-UCC | trnH-GUG, trnK-UUU^a^, trnQ-UUG, trnS-GCU, trnG-UCC^a^, trnR-UCU, trnC-GCA, trnD-GUC, trnY-GUA, trnE-UUC, trnT-GGU, trnS-UGA, trnM-CAU  trnS-GGA, trnT-UGU, trnL-UAA^a^, trnF-GAA, trnV-UAC^a^, trnM-CAU, trnW-CCA, trnP-UGG, trnI-CAU^c^, trnL-CAA^c^, trnV-GAC^c^, trnI-GAU^ac^, trnA-UGC^ac^, trnR-ACG^c^, trnN-GUU^c^, trnL-UAG, trnG-UCC | trnH-GUG, trnK-UUU^a^, trnQ-UUG, trnS-GCU, trnG-UCC^a^, trnR-UCU, trnC-GCA, trnD-GUC, trnY-GUA, trnE-UUC, trnT-GGU, trnS-UGA, trnM-CAU  trnS-GGA, trnT-UGU, trnL-UAA^a^, trnF-GAA, trnV-UAC^a^, trnM-CAU, trnW-CCA, trnP-UGG, trnI-CAU^c^, trnL-CAA^c^, trnV-GAC^c^, trnI-GAU^ac^, trnA-UGC^ac^, trnR-ACG^c^, trnN-GUU^c^, trnL-UAG, trnG-UCC | trnH-GUG, trnK-UUU^a^, trnQ-UUG, trnS-GCU, trnG-UCC^a^, trnR-UCU, trnC-GCA, trnD-GUC, trnY-GUA, trnE-UUC, trnT-GGU, trnS-UGA, trnM-CAU  trnS-GGA, trnT-UGU, trnL-UAA^a^, trnF-GAA, trnV-UAC^a^, trnM-CAU, trnW-CCA, trnP-UGG, trnI-CAU^c^, trnL-CAA^c^, trnV-GAC^c^, trnI-GAU^ac^, trnA-UGC^ac^, trnR-ACG^c^, trnN-GUU^c^, trnL-UAG, trnG-UCC | trnH-GUG, trnK-UUU^a^, trnQ-UUG, trnS-GCU, trnG-UCC^a^, trnR-UCU, trnC-GCA, trnD-GUC, trnY-GUA, trnE-UUC, trnT-GGU, trnS-UGA, trnM-CAU  trnS-GGA, trnT-UGU, trnL-UAA^a^, trnF-GAA, trnV-UAC^a^, trnM-CAU, trnW-CCA, trnP-UGG, trnI-CAU^c^, trnL-CAA^c^, trnV-GAC^c^, trnI-GAU^ac^, trnA-UGC^ac^, trnR-ACG^c^, trnN-GUU^c^, trnL-UAG, trnG-UCC | trnH-GUG, trnK-UUU^a^, trnQ-UUG, trnS-GCU, trnG-UCC^a^, trnR-UCU, trnC-GCA, trnD-GUC, trnY-GUA, trnE-UUC, trnT-GGU, trnS-UGA, trnM-CAU  trnS-GGA, trnT-UGU, trnL-UAA^a^, trnF-GAA, trnV-UAC^a^, trnM-CAU, trnW-CCA, trnP-UGG, trnI-CAU^c^, trnL-CAA^c^, trnV-GAC^c^, trnI-GAU^ac^, trnA-UGC^ac^, trnR-ACG^c^, trnN-GUU^c^, trnL-UAG, trnG-UCC | trnH-GUG, trnK-UUU^a^, trnQ-UUG, trnS-GCU, trnG-UCC^a^, trnR-UCU, trnC-GCA, trnD-GUC, trnY-GUA, trnE-UUC, trnT-GGU, trnS-UGA, trnM-CAU  trnS-GGA, trnT-UGU, trnL-UAA^a^, trnF-GAA, trnV-UAC^a^, trnM-CAU, trnW-CCA, trnP-UGG, trnI-CAU^c^, trnL-CAA^c^, trnV-GAC^c^, trnI-GAU^ac^, trnA-UGC^ac^, trnR-ACG^c^, trnN-GUU^c^, trnL-UAG, trnG-UCC | trnH-GUG, trnK-UUU^a^, trnQ-UUG, trnS-GCU, trnG-UCC^a^, trnR-UCU, trnC-GCA, trnD-GUC, trnY-GUA, trnE-UUC, trnT-GGU, trnS-UGA, trnM-CAU  trnS-GGA, trnT-UGU, trnL-UAA^a^, trnF-GAA, trnV-UAC^a^, trnM-CAU, trnW-CCA, trnP-UGG, trnI-CAU^c^, trnL-CAA^c^, trnV-GAC^c^, trnI-GAU^ac^, trnA-UGC^ac^, trnR-ACG^c^, trnN-GUU^c^, trnL-UAG, trnG-UCC | trnH-GUG, trnK-UUU^a^, trnQ-UUG, trnS-GCU, trnG-UCC^a^, trnR-UCU, trnC-GCA, trnD-GUC, trnY-GUA, trnE-UUC, trnT-GGU, trnS-UGA, trnM-CAU  trnS-GGA, trnT-UGU, trnL-UAA^a^, trnF-GAA, trnV-UAC^a^, trnM-CAU, trnW-CCA, trnP-UGG, trnI-CAU^c^, trnL-CAA^c^, trnV-GAC^c^, trnI-GAU^ac^, trnA-UGC^ac^, trnR-ACG^c^, trnN-GUU^c^, trnL-UAG, trnG-UCC | trnH-GUG, trnK-UUU^a^, trnQ-UUG, trnS-GCU, trnG-UCC^a^, trnR-UCU, trnC-GCA, trnD-GUC, trnY-GUA, trnE-UUC, trnT-GGU, trnS-UGA, trnM-CAU  trnS-GGA, trnT-UGU, trnL-UAA^a^, trnF-GAA, trnV-UAC^a^, trnM-CAU, trnW-CCA, trnP-UGG, trnI-CAU^c^, trnL-CAA^c^, trnV-GAC^c^, trnI-GAU^ac^, trnA-UGC^ac^, trnR-ACG^c^, trnN-GUU^c^, trnL-UAG, trnG-UCC | trnH-GUG, trnK-UUU^a^, trnQ-UUG, trnS-GCU, trnG-UCC^a^, trnR-UCU, trnC-GCA, trnD-GUC, trnY-GUA, trnE-UUC, trnT-GGU, trnS-UGA, trnM-CAU  trnS-GGA, trnT-UGU, trnL-UAA^a^, trnF-GAA, trnV-UAC^a^, trnM-CAU, trnW-CCA, trnP-UGG, trnI-CAU^c^, trnL-CAA^c^, trnV-GAC^c^, trnI-GAU^ac^, trnA-UGC^ac^, trnR-ACG^c^, trnN-GUU^c^, trnL-UAG, trnG-UCC | trnH-GUG, trnK-UUU^a^, trnQ-UUG, trnS-GCU, trnG-UCC^a^, trnR-UCU, trnC-GCA, trnD-GUC, trnY-GUA, trnE-UUC, trnT-GGU, trnS-UGA, trnM-CAU  trnS-GGA, trnT-UGU, trnL-UAA^a^, trnF-GAA, trnV-UAC^a^, trnM-CAU, trnW-CCA, trnP-UGG, trnI-CAU^c^, trnL-CAA^c^, trnV-GAC^c^, trnI-GAU^ac^, trnA-UGC^ac^, trnR-ACG^c^, trnN-GUU^c^, trnL-UAG, trnG-UCC |
|  | rRNA | rrn16^c^, rrn23^c^, rrn4.5^c^, rrn5^c^ | rrn16^c^, rrn23^c^, rrn4.5^c^, rrn5^c^ | rrn16^c^, rrn23^c^, rrn4.5^c^, rrn5^c^ | rrn16^c^, rrn23^c^, rrn4.5^c^, rrn5^c^ | rrn16^c^, rrn23^c^, rrn4.5^c^, rrn5^c^ | rrn16^c^, rrn23^c^, rrn4.5^c^, rrn5^c^ | rrn16^c^, rrn23^c^, rrn4.5^c^, rrn5^c^ | rrn16^c^, rrn23^c^, rrn4.5^c^, rrn5^c^ | rrn16^c^, rrn23^c^, rrn4.5^c^, rrn5^c^ | rrn16^c^, rrn23^c^, rrn4.5^c^, rrn5^c^ | rrn16^c^, rrn23^c^, rrn4.5^c^, rrn5^c^ | rrn16^c^, rrn23^c^, rrn4.5^c^, rrn5^c^ |
|  | Tanslational initiation factor | infA | infA | infA | infA | infA | infA | infA | infA | infA | infA | infA | infA |
| Photosynthetic | Subunits of photosystem I | psaB, psaA, psaI, psaJ, psaC | psaB, psaA, psaI, psaJ, psaC | psaB, psaA, psaI, psaJ, psaC | psaB, psaA, psaI, psaJ, psaC | psaB, psaA, psaI, psaJ, psaC | psaB, psaA, psaI, psaJ, psaC | psaB, psaA, psaI, psaJ, psaC | psaB, psaA, psaI, psaJ, psaC | psaB, psaA, psaI, psaJ, psaC | psaB, psaI, psaJ, psaC | psaB, psaA, psaI, psaJ, psaC | psaB, psaA, psaI, psaJ, psaC |
|  | Subunits of photosystem II | psbA, psbK, psbI, psbM, psbD, psbC, psbZ, psbJ, psbF, psbE, psbB, psbT, psbN, psbH | psbA, psbK, psbI, psbM, psbD, psbC, psbZ, psbJ, psbF, psbE, psbB, psbT, psbN, psbH | psbA, psbK, psbI, psbM, psbD, psbC, psbZ, psbJ, psbF, psbE, psbB, psbT, psbN, psbH | psbA, psbK, psbI, psbM, psbD, psbC, psbZ, psbJ, psbF, psbE, psbB, psbT, psbN, psbH | psbA, psbK, psbI, psbM, psbD, psbC, psbZ, psbJ, psbF, psbE, psbB, psbT, psbN, psbH | psbA, psbK, psbI, psbM, psbD, psbC, psbZ, psbJ, psbF, psbE, psbB, psbT, psbN, psbH | psbA, psbK, psbI, psbM, psbD, psbC, psbZ, psbJ, psbL, psbF, psbE, psbB, psbT, psbN, psbH | psbA, psbK, psbI, psbM, psbD, psbC, psbZ, psbJ, psbL, psbF, psbE, psbB, psbT, psbN, psbH | psbA, psbK, psbI, psbM, psbD, psbC, psbZ, psbJ, psbL, psbF, psbE, psbB, psbT, psbN, psbH | psbA, psbK, psbI, psbM, psbD, psbC, psbZ, psbJ, psbL, psbF, psbE, psbB, psbT, psbN, psbH | psbA, psbK, psbI, psbM, psbD, psbC, psbZ, psbJ, psbL, psbF, psbE, psbB, psbT, psbN, psbH | psbA, psbK, psbI, psbM, psbD, psbC, psbZ, psbJ, psbL, psbF, psbE, psbB, psbT, psbN, psbH |
|  | Subunits of NADH dehydrogenase | ndhJ, ndhK, ndhC, ndhB^ac^, ndhF, ndhE, ndhG, ndhI, ndhA^a^, ndhH | ndhJ, ndhK, ndhC, ndhB^ac^, ndhF^a^, ndhE, ndhG, ndhI, ndhA^a^, ndhH | ndhJ, ndhK, ndhC, ndhB^ac^, ndhF^a^, ndhE, ndhG, ndhI, ndhA^a^, ndhH | ndhJ, ndhK, ndhC, ndhB^ac^, ndhF^a^, ndhE, ndhG, ndhI, ndhA^a^, ndhH | ndhJ, ndhK, ndhC, ndhB^ac^, ndhF, ndhE, ndhG, ndhI, ndhA^a^, ndhH | ndhJ, ndhK, ndhC, ndhB^ac^, ndhF^a^, ndhE, ndhG, ndhI, ndhA^a^, ndhH | ndhJ, ndhK, ndhC, ndhB^ac^, ndhF, ndhD, ndhE, ndhG, ndhI, ndhA^a^, ndhH | ndhJ, ndhK, ndhC, ndhB^ac^, ndhF, ndhD, ndhE, ndhG, ndhI, ndhA^a^, ndhH | ndhJ, ndhK, ndhC, ndhB^ac^, ndhF^a^, ndhD, ndhE, ndhG, ndhI, ndhA^a^, ndhH | ndhJ, ndhK, ndhC, ndhB^ac^, ndhF, ndhD, ndhE, ndhG, ndhI, ndhA^a^, ndhH | ndhJ, ndhK, ndhC, ndhB^ac^, ndhF, ndhD, ndhE, ndhG, ndhI, ndhA^a^, ndhH | ndhJ, ndhK, ndhC, ndhB^ac^, ndhF, ndhD, ndhE, ndhG, ndhI, ndhA^a^, ndhH |
|  | Subunits of cytochrome b/f complex | petN, petA, petL, petG, petB^a^, petD^a^ | petN, petA, petL, petG, petB, petD^a^ | petN, petA, petL, petG, petB, petD^a^ | petN, petA, petL, petG, petB, petD^a^ | petN, petA, petL, petG, petB^a^, petD^a^ | petN, petA, petL, petG, petB, petD^a^ | petN, petA, petL, petG, petB^a^, petD^a^ | petN, petA, petL, petG, petB^a^, petD^a^ | petN, petA, petL, petG, petB^a^, petD^a^ | petN, petA, petL, petG, petB^a^, petD^a^ | petN, petA, petL, petG, petB^a^, petD^a^ | petN, petA, petL, petG, petB^a^, petD^a^ |
|  | Subunits of ATP synthase | atpA, atpF^a^, atpH, atpI, atpE, atpB | atpA, atpF^a^, atpH, atpI, atpE, atpB | atpA, atpF^a^, atpH, atpI, atpE, atpB | atpA, atpF^a^, atpH, atpI, atpE, atpB | atpA, atpF^a^, atpH, atpI, atpE, atpB | atpA, atpF^a^, atpH, atpI, atpE, atpB | atpA, atpF^a^, atpH, atpI, atpE, atpB | atpA, atpF^a^, atpH, atpI, atpE, atpB | atpA, atpF^a^, atpH, atpI, atpE, atpB | atpA, atpF^a^, atpH, atpI, atpE, atpB | atpA, atpF^a^, atpH, atpI, atpE, atpB | atpA, atpF^a^, atpH, atpI, atpE, atpB |
|  | Large subunit of RubisCO | rbcL | rbcL | rbcL | rbcL | rbcL | rbcL | rbcL | rbcL | rbcL | rbcL | rbcL | rbcL |
| Other | Protease | clpP^a^ | clpP^a^ | clpP^a^ | clpP^a^ | clpP^a^ | clpP^a^ | clpP^a^ | clpP^a^ | clpP^a^ | clpP^a^ | clpP^a^ | clpP^a^ |
|  | Maturase | matK | matK | matK | matK | matK | matK | matK | matK | matK | matK | matK | matK |
|  | Envelope membrane protein | cemA | cemA | cemA | cemA | cemA | cemA | cemA | cemA | cemA | cemA | cemA | cemA |
|  | c-type cytochrome synthesis gene | ccsA | ccsA | ccsA | ccsA | ccsA | ccsA | ccsA | ccsA | ccsA | ccsA | ccsA | ccsA |
|  | Subunit of Acetyl-CoA-carboxylase | accD | accD | accD | accD | accD | accD | accD | accD | accD | accD | accD | accD |
|  | Hypotheticalch loroplast reading frames | ycf3^a^, ycf4, ycf2^c^, ycf1^c^ | ycf3^a^, ycf4, ycf2^c^, ycf1^c^ | ycf3^a^, ycf4, ycf2^c^, ycf1^c^ | ycf3^a^, ycf4, ycf2^c^ | ycf3^a^, ycf4, ycf2^c^, ycf1^c^ | ycf3^a^, ycf4, ycf2^c^, ycf1^c^ | ycf3^a^, ycf4, ycf2^c^, ycf1^c^ | ycf3^a^, ycf4, ycf2^c^, ycf1^c^ | ycf3^a^, ycf4, ycf2^c^, ycf1^c^ | ycf3^a^, ycf4, ycf2^c^, ycf1^c^ | ycf3^a^, ycf4, ycf2^c^, ycf1^c^ | ycf3^a^, ycf4, ycf2^c^, ycf1^c^ |
| Number of repeated genes | | 11 | 18 | 18 | 17 | 18 | 18 | 18 | 18 | 18 | 18 | 18 | 18 |
| Number of protein genes | | 74 | 76 | 76 | 75 | 74 | 76 | 78 | 78 | 78 | 77 | 76 | 76 |
| Number of tRNA | | 29 | 30 | 30 | 30 | 30 | 30 | 30 | 30 | 30 | 30 | 30 | 30 |
| Number of rRNA | | 4 | 4 | 4 | 4 | 4 | 4 | 4 | 4 | 4 | 4 | 4 | 4 |

a Indicate genes containing introns, c indicates that the gene has two copies.

*The sample was collected from Xing'an County in Guilin (GenBank No. OR570614)

#The sample was collected from Sanjiang County in Liuzhou (GenBank No. OR570615)
