# Supplementary material for: Chloroplast genomic characterization and phylogenetic analysis of eleven Persicaria medicinal plants from Guangxi, Southern China
Source: Front Plant Sci. 2026 Jan 22;16:1749088. doi: 10.3389/fpls.2025.1749088 (PMC12872838; doi:10.3389/fpls.2025.1749088)
Supplement: Supplementary file 3 [file Table3.docx]

Table S3 Codon-anticodon recognition patterns and codon usage of the 11 *Persicaria* species

| Amino Acid | Codon | *P. capitata* | | *P. glabra* | | *P. pubescens* | | *P. tinctoria* | | *P. chinensis* | | *P. longiseta* | | *P. maackiana* | | *P. hastatosagittata* | | *P. lapathifolia* | | *P. perfoliata* | | *P. hydropiper** | | *P. hydropiper#* | |
| --- | --- | --- | --- | --- | --- | --- | --- | --- | --- | --- | --- | --- | --- | --- | --- | --- | --- | --- | --- | --- | --- | --- | --- | --- | --- |
|  |  | Count | RSCU | Count | RSCU | Count | RSCU | Count | RSCU | Count | RSCU | Count | RSCU | Count | RSCU | Count | RSCU | Count | RSCU | Count | RSCU | Count | RSCU | Count | RSCU |
| ‌Leu | CTA | 374 | 0.803 | 395 | 0.831 | 394 | 0.829 | 348 | 0.843 | 376 | 0.806 | 388 | 0.83 | 411 | 0.837 | 400 | 0.819 | 408 | 0.833 | 395 | 0.829 | 403 | 0.828 | 403 | 0.829 |
|  | CTC | 196 | 0.421 | 201 | 0.423 | 198 | 0.417 | 165 | 0.4 | 194 | 0.416 | 191 | 0.409 | 208 | 0.423 | 205 | 0.42 | 206 | 0.42 | 206 | 0.432 | 202 | 0.415 | 202 | 0.416 |
|  | CTG | 188 | 0.403 | 193 | 0.406 | 194 | 0.408 | 158 | 0.383 | 190 | 0.407 | 184 | 0.394 | 191 | 0.389 | 193 | 0.395 | 196 | 0.4 | 184 | 0.386 | 198 | 0.407 | 198 | 0.407 |
|  | CTT | 610 | 1.309 | 628 | 1.321 | 625 | 1.316 | 544 | 1.318 | 611 | 1.31 | 619 | 1.324 | 634 | 1.291 | 625 | 1.28 | 646 | 1.318 | 610 | 1.28 | 634 | 1.303 | 634 | 1.305 |
|  | TTA | 840 | 1.803 | 824 | 1.733 | 826 | 1.739 | 726 | 1.759 | 841 | 1.803 | 816 | 1.745 | 880 | 1.792 | 898 | 1.84 | 861 | 1.757 | 875 | 1.836 | 862 | 1.771 | 859 | 1.767 |
|  | TTG | 588 | 1.262 | 612 | 1.287 | 613 | 1.291 | 535 | 1.296 | 587 | 1.258 | 607 | 1.298 | 623 | 1.268 | 608 | 1.245 | 623 | 1.271 | 589 | 1.236 | 621 | 1.276 | 620 | 1.276 |
| ‌Ile | ATA | 715 | 0.96 | 713 | 0.947 | 715 | 0.952 | 596 | 0.918 | 716 | 0.962 | 712 | 0.959 | 763 | 0.974 | 770 | 0.973 | 747 | 0.959 | 726 | 0.949 | 741 | 0.962 | 741 | 0.963 |
|  | ATC | 398 | 0.534 | 420 | 0.558 | 418 | 0.556 | 364 | 0.561 | 398 | 0.534 | 415 | 0.559 | 419 | 0.535 | 415 | 0.525 | 428 | 0.549 | 408 | 0.534 | 419 | 0.544 | 419 | 0.544 |
|  | ATT | 1122 | 1.506 | 1125 | 1.495 | 1121 | 1.492 | 987 | 1.521 | 1120 | 1.504 | 1101 | 1.482 | 1169 | 1.492 | 1188 | 1.502 | 1162 | 1.492 | 1160 | 1.517 | 1151 | 1.494 | 1149 | 1.493 |
| ‌Ser | AGC | 139 | 0.424 | 147 | 0.442 | 150 | 0.45 | 125 | 0.435 | 140 | 0.428 | 143 | 0.436 | 151 | 0.437 | 147 | 0.43 | 150 | 0.438 | 145 | 0.435 | 148 | 0.435 | 148 | 0.435 |
|  | AGT | 384 | 1.171 | 386 | 1.161 | 386 | 1.157 | 328 | 1.142 | 384 | 1.173 | 376 | 1.145 | 403 | 1.168 | 398 | 1.163 | 395 | 1.152 | 382 | 1.145 | 394 | 1.158 | 394 | 1.158 |
|  | TCA | 378 | 1.152 | 381 | 1.146 | 382 | 1.145 | 320 | 1.114 | 380 | 1.161 | 378 | 1.151 | 398 | 1.153 | 400 | 1.169 | 398 | 1.161 | 393 | 1.178 | 394 | 1.158 | 393 | 1.155 |
|  | TCC | 314 | 0.957 | 332 | 0.998 | 329 | 0.987 | 297 | 1.034 | 313 | 0.956 | 323 | 0.984 | 328 | 0.95 | 327 | 0.956 | 337 | 0.983 | 322 | 0.965 | 336 | 0.987 | 335 | 0.985 |
|  | TCG | 205 | 0.625 | 199 | 0.598 | 197 | 0.591 | 169 | 0.588 | 203 | 0.62 | 196 | 0.597 | 212 | 0.614 | 210 | 0.614 | 205 | 0.598 | 196 | 0.587 | 202 | 0.594 | 203 | 0.597 |
|  | TCT | 548 | 1.671 | 550 | 1.654 | 557 | 1.67 | 485 | 1.688 | 544 | 1.662 | 554 | 1.687 | 579 | 1.677 | 571 | 1.669 | 572 | 1.668 | 564 | 1.69 | 568 | 1.669 | 568 | 1.67 |
| Gly | GGA | 663 | 1.523 | 673 | 1.512 | 674 | 1.515 | 611 | 1.489 | 663 | 1.522 | 659 | 1.504 | 712 | 1.549 | 715 | 1.558 | 700 | 1.523 | 699 | 1.58 | 696 | 1.526 | 696 | 1.526 |
|  | GGC | 219 | 0.503 | 230 | 0.517 | 225 | 0.506 | 224 | 0.546 | 220 | 0.505 | 228 | 0.52 | 226 | 0.492 | 226 | 0.492 | 235 | 0.511 | 214 | 0.484 | 232 | 0.509 | 232 | 0.509 |
|  | GGG | 318 | 0.731 | 328 | 0.737 | 327 | 0.735 | 298 | 0.726 | 320 | 0.735 | 326 | 0.744 | 326 | 0.709 | 323 | 0.704 | 337 | 0.733 | 312 | 0.705 | 332 | 0.728 | 332 | 0.728 |
|  | GGT | 541 | 1.243 | 549 | 1.234 | 553 | 1.243 | 508 | 1.238 | 539 | 1.238 | 540 | 1.232 | 575 | 1.251 | 572 | 1.246 | 567 | 1.233 | 545 | 1.232 | 564 | 1.237 | 564 | 1.237 |
| Arg | AGA | 488 | 1.726 | 488 | 1.689 | 495 | 1.711 | 400 | 1.621 | 488 | 1.729 | 485 | 1.69 | 501 | 1.694 | 504 | 1.708 | 502 | 1.706 | 500 | 1.713 | 498 | 1.703 | 498 | 1.703 |
|  | AGG | 223 | 0.789 | 228 | 0.789 | 228 | 0.788 | 185 | 0.749 | 220 | 0.78 | 226 | 0.787 | 226 | 0.764 | 226 | 0.766 | 235 | 0.798 | 223 | 0.764 | 234 | 0.8 | 234 | 0.8 |
|  | CGA | 402 | 1.422 | 410 | 1.419 | 399 | 1.379 | 339 | 1.373 | 403 | 1.428 | 398 | 1.387 | 415 | 1.404 | 406 | 1.376 | 415 | 1.41 | 408 | 1.398 | 404 | 1.381 | 404 | 1.381 |
|  | CGC | 108 | 0.382 | 102 | 0.353 | 104 | 0.359 | 102 | 0.413 | 107 | 0.379 | 108 | 0.376 | 113 | 0.382 | 113 | 0.383 | 105 | 0.357 | 107 | 0.367 | 106 | 0.362 | 106 | 0.362 |
|  | CGG | 117 | 0.414 | 136 | 0.471 | 140 | 0.484 | 125 | 0.506 | 117 | 0.415 | 137 | 0.477 | 140 | 0.474 | 142 | 0.481 | 138 | 0.469 | 141 | 0.483 | 143 | 0.489 | 144 | 0.492 |
|  | CGT | 358 | 1.267 | 370 | 1.28 | 370 | 1.279 | 330 | 1.337 | 358 | 1.269 | 368 | 1.282 | 379 | 1.282 | 379 | 1.285 | 371 | 1.26 | 372 | 1.275 | 370 | 1.265 | 369 | 1.262 |
| ‌Phe | TTC | 496 | 0.644 | 534 | 0.686 | 536 | 0.685 | 458 | 0.693 | 497 | 0.645 | 512 | 0.673 | 514 | 0.645 | 518 | 0.65 | 551 | 0.686 | 501 | 0.654 | 548 | 0.685 | 547 | 0.686 |
|  | TTT | 1044 | 1.356 | 1023 | 1.314 | 1029 | 1.315 | 864 | 1.307 | 1045 | 1.355 | 1010 | 1.327 | 1079 | 1.355 | 1077 | 1.35 | 1056 | 1.314 | 1030 | 1.346 | 1051 | 1.315 | 1048 | 1.314 |
| ‌Lys | AAA | 1136 | 1.48 | 1164 | 1.488 | 1169 | 1.489 | 876 | 1.487 | 1130 | 1.472 | 1179 | 1.503 | 1203 | 1.495 | 1204 | 1.488 | 1182 | 1.484 | 1189 | 1.49 | 1185 | 1.488 | 1185 | 1.488 |
|  | AAG | 399 | 0.52 | 400 | 0.512 | 401 | 0.511 | 302 | 0.513 | 405 | 0.528 | 390 | 0.497 | 406 | 0.505 | 414 | 0.512 | 411 | 0.516 | 407 | 0.51 | 408 | 0.512 | 408 | 0.512 |
| Glu | GAA | 1075 | 1.454 | 1105 | 1.457 | 1105 | 1.457 | 889 | 1.441 | 1069 | 1.45 | 1099 | 1.454 | 1110 | 1.451 | 1107 | 1.457 | 1126 | 1.461 | 1104 | 1.465 | 1128 | 1.464 | 1128 | 1.464 |
|  | GAG | 404 | 0.546 | 412 | 0.543 | 412 | 0.543 | 345 | 0.559 | 405 | 0.55 | 413 | 0.546 | 420 | 0.549 | 413 | 0.543 | 415 | 0.539 | 403 | 0.535 | 413 | 0.536 | 413 | 0.536 |
| Val | GTA | 522 | 1.437 | 533 | 1.459 | 528 | 1.446 | 474 | 1.455 | 521 | 1.437 | 518 | 1.447 | 544 | 1.459 | 548 | 1.46 | 537 | 1.445 | 530 | 1.457 | 533 | 1.434 | 533 | 1.434 |
|  | GTC | 205 | 0.564 | 218 | 0.597 | 220 | 0.602 | 190 | 0.583 | 205 | 0.566 | 214 | 0.598 | 220 | 0.59 | 214 | 0.57 | 223 | 0.6 | 207 | 0.569 | 225 | 0.605 | 225 | 0.605 |
|  | GTG | 195 | 0.537 | 197 | 0.539 | 197 | 0.539 | 173 | 0.531 | 196 | 0.541 | 193 | 0.539 | 197 | 0.529 | 199 | 0.53 | 197 | 0.53 | 187 | 0.514 | 197 | 0.53 | 197 | 0.53 |
|  | GTT | 531 | 1.462 | 513 | 1.405 | 516 | 1.413 | 466 | 1.431 | 528 | 1.457 | 507 | 1.416 | 530 | 1.422 | 540 | 1.439 | 530 | 1.426 | 531 | 1.46 | 532 | 1.431 | 532 | 1.431 |
| Ala | GCA | 403 | 1.131 | 410 | 1.129 | 407 | 1.121 | 378 | 1.103 | 402 | 1.128 | 404 | 1.117 | 417 | 1.125 | 406 | 1.096 | 419 | 1.129 | 396 | 1.119 | 418 | 1.127 | 418 | 1.127 |
|  | GCC | 252 | 0.707 | 263 | 0.724 | 265 | 0.73 | 244 | 0.712 | 253 | 0.71 | 256 | 0.708 | 246 | 0.664 | 252 | 0.68 | 270 | 0.727 | 246 | 0.695 | 269 | 0.725 | 269 | 0.726 |
|  | GCG | 174 | 0.488 | 185 | 0.509 | 189 | 0.521 | 181 | 0.528 | 176 | 0.494 | 189 | 0.522 | 195 | 0.526 | 200 | 0.54 | 189 | 0.509 | 184 | 0.52 | 194 | 0.523 | 193 | 0.521 |
|  | GCT | 596 | 1.673 | 595 | 1.638 | 591 | 1.628 | 568 | 1.657 | 595 | 1.669 | 598 | 1.653 | 625 | 1.686 | 624 | 1.684 | 607 | 1.635 | 590 | 1.667 | 603 | 1.625 | 603 | 1.626 |
| Thr | ACA | 412 | 1.259 | 437 | 1.289 | 433 | 1.279 | 363 | 1.229 | 409 | 1.253 | 417 | 1.258 | 432 | 1.234 | 434 | 1.249 | 442 | 1.27 | 423 | 1.257 | 439 | 1.265 | 439 | 1.267 |
|  | ACC | 237 | 0.724 | 234 | 0.69 | 234 | 0.691 | 205 | 0.694 | 237 | 0.726 | 227 | 0.685 | 242 | 0.691 | 242 | 0.696 | 244 | 0.701 | 230 | 0.684 | 242 | 0.697 | 242 | 0.698 |
|  | ACG | 165 | 0.504 | 177 | 0.522 | 179 | 0.529 | 147 | 0.498 | 161 | 0.493 | 179 | 0.54 | 195 | 0.557 | 187 | 0.538 | 183 | 0.526 | 179 | 0.532 | 184 | 0.53 | 183 | 0.528 |
|  | ACT | 495 | 1.513 | 508 | 1.499 | 508 | 1.501 | 466 | 1.578 | 499 | 1.528 | 503 | 1.517 | 531 | 1.517 | 527 | 1.517 | 523 | 1.503 | 514 | 1.527 | 523 | 1.507 | 522 | 1.506 |
| Asn | AAC | 300 | 0.488 | 316 | 0.496 | 312 | 0.491 | 271 | 0.51 | 300 | 0.489 | 305 | 0.489 | 318 | 0.496 | 331 | 0.509 | 324 | 0.498 | 321 | 0.507 | 317 | 0.492 | 317 | 0.493 |
|  | AAT | 929 | 1.512 | 958 | 1.504 | 960 | 1.509 | 792 | 1.49 | 928 | 1.511 | 943 | 1.511 | 965 | 1.504 | 970 | 1.491 | 978 | 1.502 | 946 | 1.493 | 971 | 1.508 | 969 | 1.507 |
| Pro | CCA | 305 | 1.062 | 308 | 1.066 | 307 | 1.06 | 268 | 1.06 | 302 | 1.053 | 304 | 1.059 | 310 | 1.036 | 304 | 1.016 | 318 | 1.072 | 301 | 1.033 | 314 | 1.066 | 314 | 1.067 |
|  | CCC | 223 | 0.776 | 238 | 0.824 | 237 | 0.819 | 205 | 0.811 | 228 | 0.795 | 233 | 0.812 | 237 | 0.792 | 235 | 0.785 | 243 | 0.819 | 234 | 0.803 | 240 | 0.815 | 240 | 0.816 |
|  | CCG | 177 | 0.616 | 172 | 0.595 | 174 | 0.601 | 156 | 0.617 | 179 | 0.624 | 175 | 0.61 | 191 | 0.638 | 195 | 0.652 | 176 | 0.593 | 178 | 0.611 | 175 | 0.594 | 174 | 0.591 |
|  | CCT | 444 | 1.546 | 438 | 1.516 | 440 | 1.52 | 382 | 1.511 | 438 | 1.527 | 436 | 1.519 | 459 | 1.534 | 463 | 1.547 | 450 | 1.516 | 452 | 1.552 | 449 | 1.525 | 449 | 1.526 |
| Asp | GAC | 218 | 0.398 | 227 | 0.408 | 224 | 0.404 | 189 | 0.407 | 220 | 0.4 | 226 | 0.409 | 236 | 0.42 | 237 | 0.42 | 234 | 0.414 | 230 | 0.418 | 227 | 0.402 | 227 | 0.403 |
|  | GAT | 877 | 1.602 | 886 | 1.592 | 886 | 1.596 | 740 | 1.593 | 880 | 1.6 | 879 | 1.591 | 889 | 1.58 | 891 | 1.58 | 896 | 1.586 | 871 | 1.582 | 901 | 1.598 | 900 | 1.597 |
| Tyr | TAC | 199 | 0.412 | 205 | 0.412 | 208 | 0.42 | 168 | 0.403 | 200 | 0.414 | 202 | 0.414 | 212 | 0.409 | 214 | 0.411 | 215 | 0.418 | 211 | 0.415 | 212 | 0.416 | 212 | 0.417 |
|  | TAT | 768 | 1.588 | 789 | 1.588 | 783 | 1.58 | 665 | 1.597 | 767 | 1.586 | 775 | 1.586 | 824 | 1.591 | 827 | 1.589 | 813 | 1.582 | 806 | 1.585 | 807 | 1.584 | 804 | 1.583 |
| Gln | CAA | 680 | 1.495 | 715 | 1.494 | 719 | 1.498 | 610 | 1.486 | 680 | 1.495 | 705 | 1.495 | 714 | 1.511 | 710 | 1.507 | 730 | 1.502 | 694 | 1.51 | 728 | 1.504 | 728 | 1.506 |
|  | CAG | 230 | 0.505 | 242 | 0.506 | 241 | 0.502 | 211 | 0.514 | 230 | 0.505 | 238 | 0.505 | 231 | 0.489 | 232 | 0.493 | 242 | 0.498 | 225 | 0.49 | 240 | 0.496 | 239 | 0.494 |
| His | CAC | 152 | 0.512 | 147 | 0.484 | 142 | 0.473 | 131 | 0.488 | 150 | 0.502 | 140 | 0.469 | 163 | 0.523 | 157 | 0.502 | 149 | 0.477 | 141 | 0.487 | 143 | 0.467 | 143 | 0.467 |
|  | CAT | 442 | 1.488 | 460 | 1.516 | 459 | 1.527 | 406 | 1.512 | 448 | 1.498 | 457 | 1.531 | 460 | 1.477 | 468 | 1.498 | 476 | 1.523 | 438 | 1.513 | 469 | 1.533 | 469 | 1.533 |
| Met | ATG | 540 | 1 | 557 | 1 | 560 | 1 | 456 | 1 | 538 | 1 | 540 | 1 | 577 | 1 | 574 | 1 | 588 | 1 | 566 | 1 | 576 | 1 | 575 | 1 |
| ‌Trp | TGG | 477 | 1 | 483 | 1 | 484 | 1 | 425 | 1 | 479 | 1 | 481 | 1 | 489 | 1 | 492 | 1 | 491 | 1 | 461 | 1 | 493 | 1 | 493 | 1 |
| Cys | TGC | 84 | 0.566 | 91 | 0.603 | 90 | 0.604 | 75 | 0.562 | 85 | 0.572 | 85 | 0.576 | 84 | 0.551 | 83 | 0.544 | 91 | 0.597 | 83 | 0.55 | 89 | 0.595 | 89 | 0.595 |
|  | TGT | 213 | 1.434 | 211 | 1.397 | 208 | 1.396 | 192 | 1.438 | 212 | 1.428 | 210 | 1.424 | 221 | 1.449 | 222 | 1.456 | 214 | 1.403 | 219 | 1.45 | 210 | 1.405 | 210 | 1.405 |

*The sample was collected from Xing'an County in Guilin (GenBank No. OR570614)

#The sample was collected from Sanjiang County in Liuzhou (GenBank No. OR570615)
